# Role of photolyase in stress tolerance and virulence of plant-pathogenic bacterium *Pseudomonas cichorii* JBC1

**DOI:** 10.1128/aem.00255-26

**Published:** 2026-05-12

**Authors:** Nguyen Van Khanh, Shamjetsabam Gangarani Devi, Yong Hoon Lee

**Affiliations:** 1 Division of Biotechnology, Jeonbuk National University 26714, Iksan-si, Republic of Korea; 2 Advanced Institute of Environment and Bioscience, Plant Medical Research Center, Jeonbuk National University 26714, Jeonju-si, Republic of Korea; The University of Tennessee Knoxville, Knoxville, Tennessee, USA

**Keywords:** photolyase, photoreactivation, *Pseudomonas cichorii *JBC1, stress tolerance, survival, virulence

## Abstract

The cryptochrome/photolyase family (CPF) comprises structurally conserved flavoproteins with diverse roles in light-dependent DNA repair and stress adaptation. Here, we identified and functionally characterized a class I cyclobutane pyrimidine dimer (CPD) photolyase, Pc-Phr, from the plant-pathogenic bacterium *Pseudomonas cichorii* JBC1 (PcJBC1). Spectroscopic analyses revealed that Pc-Phr binds flavin adenine dinucleotide (FAD) and undergoes characteristic blue light–induced photoreduction from the oxidized state (FAD_OX_) to catalytically active reduced forms. Pc-Phr exhibited robust photoreactivation activity, repairing UV-induced CPDs *in vitro* and restoring the viability of UV-C–irradiated cells *in vivo*, whereas this activity was abolished in a *phr* deletion mutant. Transcriptional analyses showed that *phr* expression is induced by UV-C and further enhanced by blue light, consistent with a light-dependent DNA damage response. Beyond DNA repair, Pc-Phr promoted bacterial survival, growth, and adhesion under UV-C, high-intensity blue light, and oxidative stress conditions, and was essential for maintaining intracellular reactive oxygen species (ROS) homeostasis. Deletion of *phr* resulted in a drastic ROS accumulation and significantly reduced survival under both UV-C and H_2_O_2_ stress. Importantly, Pc-Phr contributed to virulence in host plants, with the mutant exhibiting markedly reduced pathogenicity. These results demonstrate that efficient photorepair is a critical determinant of environmental fitness and pathogenicity in *P. cichorii*, highlighting the importance of light-responsive DNA repair systems in plant-associated bacteria.

**IMPORTANCE:**

Plant-pathogenic bacteria must withstand environmental stresses such as ultraviolet radiation and host-derived oxidative bursts to survive and cause disease. Although photolyases are well known for repairing UV-induced DNA lesions, their broader roles in bacterial physiology and pathogenicity remain insufficiently understood. This study demonstrates that the photolyase Pc-Phr in *Pseudomonas cichorii* not only facilitates efficient repair of UV-induced DNA but also plays a critical role in maintaining intracellular redox balance. Loss of Pc-Phr led to excessive accumulation of ROS, increased sensitivity to environmental stress, and reduced bacterial fitness. Importantly, Pc-Phr contributes to virulence-associated traits, including adhesion to host surfaces, colonization, and survival in planta, revealing a direct link between photoreactivation, oxidative stress tolerance, and pathogenicity. These findings expand the functional scope of photolyases beyond genome maintenance and reveal their significance in environmental adaptation and plant-microbe interactions.

## INTRODUCTION

Light is a critical environmental signal that influences diverse physiological and behavioral processes in bacteria, including growth, metabolite production, and stress responses, as well as multicellular behaviors, such as swarming motility and biofilm formation (1). Among various wavelengths, ultraviolet (UV) radiation is particularly harmful due to its ability to induce molecular damage, especially to nucleic acids. If not repaired, UV-induced DNA lesions can lead to genomic instability, mutagenesis, or cell death (2, 3). The most prevalent forms of UV-induced DNA damage are cyclobutane pyrimidine dimers (CPDs) and pyrimidine–pyrimidone (6–4) photoproducts ((6–4)PPs), which account for approximately 80%–90% and 10%–20% of total lesions, respectively (4–6).

To counteract the deleterious effects, many organisms have evolved dedicated DNA repair mechanisms. Among these, photoreactivation mediated by photolyases (Phrs), a subclass of the cryptochrome/photolyase family (CPF), is one of the most efficient and evolutionarily conserved mechanisms. Phrs catalyze the light-dependent repair of DNA lesions, particularly CPDs and (6–4)PPs, using blue-to-near-UV light (300–500 nm) as an energy source (7, 8). Phrs are broadly distributed across bacteria, fungi, plants, and animals (8). Phrs contain and utilize flavin adenine dinucleotide (FAD) as a catalytic cofactor. Additionally, they employ an auxiliary chromophore, either 5,10-methenyltetrahydrofolate (MTHF) or 8-hydroxy-7,8-didemethyl-5-deaza-riboflavin (8-HDF), to enhance light absorption (9, 10).

Based on substrate specificity, Phrs are classified into CPD and (6–4) photolyases (11, 12). CPD photolyases are further subdivided into classes I, II, III, and Cry-DASH based on sequence homology and phylogenetic relationships (10). Recent phylogenetic analyses have significantly expanded the diversity of CPF, particularly among prokaryotes. These include iron–sulfur bacterial cryptochromes and photolyases (FeS-BCPs), a distinct class of (6–4) photolyases harboring a [4Fe–4S] cluster and 6,7-dimethyl-8-ribityllumazine (DLZ) as an additional chromophore (7, 13), as well as newly described short photolyase-like proteins (NewPHLs or SPLs), which repair CPDs in single-stranded DNA and exhibit an inverted domain topology (14). In contrast, cryptochromes (Crys), despite sharing high structural similarity with Phrs, have lost DNA repair activity and function instead as blue/UV-A photoreceptors that regulate signaling pathways governing growth, development, and circadian rhythms in eukaryotes (3, 8, 15).

Approximately half of the bacterial species analyzed to date possess Phr homologs, suggesting widespread conservation of photoreactivation mechanisms among prokaryotes (16). The first Phr was purified and characterized from *Escherichia coli* (17). Subsequent studies have identified and characterized Phrs from various prokaryotes, including *Thermus thermophilus* (18), *P. aeruginosa,* and *P. syringae* (19), *Shewanella oneidensis* (20), *P. syringae* pv. *syringae* B728a (21), and *Agrobacterium tumefaciens* (22). Functional analyses of Phrs have demonstrated that photoreactivation significantly enhances bacterial survival both under UV-B (19–21) and blue light (BL) (23) stress conditions.

Plant-pathogenic bacteria encounter multiple environmental and host-derived stresses throughout their life cycle, which directly influence their survival, colonization, and disease development. In addition to abiotic factors, such as nutrient limitation, temperature extremes, salinity, drought, and solar radiation, plant pathogens must overcome host immune responses during infection. UV radiation not only induces DNA damage but also promotes the generation of reactive oxygen species (ROS), exacerbating cellular stress (24). During pathogen recognition, plant cells rapidly produce ROS, including hydrogen peroxide (H_2_O_2_) and superoxide radicals, which exert antimicrobial effects by damaging bacterial DNA, proteins, and membranes (25). To persist and cause disease, bacterial pathogens must therefore deploy coordinated protective mechanisms, including DNA repair systems and defenses against oxidative stress. Previous studies have shown that photolyases contribute to oxidative stress tolerance in bacteria such as *Neisseria gonorrhoeae* and *Vibrio cholerae*, linking photorepair capacity to broader stress adaptation (23, 26).

*Pseudomonas cichorii* JBC1 (PcJBC1), a gram-negative bacterium, is pathogenic to a wide range of vegetables and crops (27, 28). Although genomic analyses have identified a putative CPF member in PcJBC1, its biochemical properties and physiological roles remain unexplored. In this study, we functionally characterized a putative class I CPD photolyase, Pc-Phr, from *P. cichorii* JBC1. Using biochemical, genetic, and physiological approaches, we investigated its role in photoreactivation, stress tolerance, and pathogenicity. We demonstrate that Pc-Phr is a light-responsive DNA repair enzyme that enhances bacterial survival under photic and oxidative stress conditions and contributes to virulence *in planta*. Our results provide new insight into how light-dependent DNA repair mechanisms support environmental fitness and pathogenicity in plant-associated bacteria.

## MATERIALS AND METHODS

### Phr gene identification and domain analysis

Conserved domains of the putative Phr/Cry protein (Pc-Phr, NCBI protein accession no. AHF66055.1) were identified in the genome (NCBI accession no. CP007039.1) of PcJBC1 (28) using the Conserved Domains Database (CDD) tool (29). The functional domain architecture was further analyzed using the SMART database (30). The deduced amino acid sequence of Pc-Phr was aligned with over 50 characterized Phr and Cry sequences from multiple kingdoms, including bacteria, Archaea, cyanobacteria, algae, yeast, fungi, plants, and animals, as retrieved from the NCBI and UniProt databases. Multiple sequence alignment was performed using Clustal Omega (31) via the European Bioinformatics Institute. Phylogenetic analysis was conducted using MEGA11 (32) based on the neighbor-joining method with 1,000 bootstrap replicates, and the resulting unrooted tree was visualized.

Representative sequences included class I CPD photolyases from *Pseudomonas koreensis* (NCBI accession no. RVD74662), *V. cholerae* O1 (Q9KNA8), and *Escherichia coli* K-12 (P00914); class II CPD Phrs from *Arabidopsis thaliana* (Q9SB00), *Chlorobium ferrooxidans* DSM13031 (Q0YV00), and *Methanosarcina mazei* (Q8PYK9); class III CPD Phrs from *Agrobacterium fabrum* C58 (AAK87020), *Azorhizobium caulinodans* ORS 571 (BAF88822), and *Bradyrhizobium diazoefficiens* USDA 110 (BAC50575); and (6–4)Phrs from *Drosophila melanogaster* (Q24281), *Oryza sativa* (Q0E2Y1), and *Gloeobacter violaceus* (Q7NJT3). Cry-DASH proteins from *Gloeobacter violaceus* PCC7421 (Q7NMD1), *Synechocystis* sp. PCC6803 (P77967), and *Ostreococcus tauri* (Q5IFN2); iron–sulfur bacterial cryptochromes and photolyases (FeS-BCPs) from *V. cholerae* (UniProt Q9KLD7), *Cereibacter sphaeroides* (Q3IXP1), and *Agrobacterium fabrum* (A9CH39); and NewPHLs from *Dinoroseobacter shibae* (A8LJA9), *Methylobacterium mesophilicum* (M7YZC8), *Erythrobacter litoralis* (Q2N8F4), and *Rhodospirillum centenum* (B6IPR5) were also included. Physicochemical properties of Pc-Phr, including molecular weight (MW, kDa), isoelectric point (pI), and grand average hydropathy value (GRAVY), were predicted using the ExPaSy-Protparam tool (33).

### Overexpression of Pc-Phr

The *phr* gene was amplified from the PcJBC1 genomic DNA using primer sets HindIII-PHR-F and BamHI-PHR-R (Table S1) and then cloned into the pET-28a vector with *Hind*III and *Bam*HI restriction sites. The recombinant plasmid, pET-28a:phr, was transformed into *E. coli* TOP10 cells. The transformants were screened on Luria–Bertani (LB) agar containing kanamycin (50 μg/mL) and verified by the polymerase chain reaction (PCR) method and sequencing using primers T7_promoter_F and T7_terminator_R. For protein expression, *E. coli* BL21 cells were transformed with pET-28a:phr and grown overnight at 37°C in LB medium supplemented with kanamycin (50 μg/mL) at 180 rpm. The overnight bacterial culture (2%, vol/vol) was seeded into 500 mL fresh LB medium containing riboflavin (10 µM) and kanamycin (50 μg/mL). The bacteria were cultured at 37°C until a specific optical density at 600 nm (OD_600_) of 0.5–0.8 was reached. Pc-Phr was expressed with 0.3 mM isopropyl-β-D-thiogalactopyranoside, and the culture was incubated at 12°C for 24 h in the dark. Cells were harvested by centrifugation (10,000 rpm, 10 min, 4°C) and stored at −20°C for further analysis.

### Purification of Pc-Phr

Recombinant Pc-Phr was purified using the method described by Goett-Zink et al. (34) with minor modifications. Briefly, the overexpressed cells were thawed and resuspended in a 50 mM phosphate buffer (pH 8.0) containing 300 mM NaCl, 10 mM imidazole, 20% glycerol, and phenylmethylsulfonyl fluoride (Sigma-Aldrich). The cells were lysed using a sonic dismembrator (30% amplitude, 2 s pulse/2 s pause, 4 min/cycle, 3 cycles). The lysate was centrifuged at 14,000 rpm at 4°C for 15 min, and the supernatant was loaded into a HisTrap FF column (GE Healthcare, USA). After washing with 20 column volumes of wash buffer, Pc-Phr was eluted using a stepwise imidazole gradient (50–150 mM). Fractions containing Pc-Phr were identified by SDS-PAGE, pooled, and buffer-exchanged into 50 mM phosphate buffer (pH 8.0), containing 100 mM NaCl and 20% glycerol using a 10-kDa cutoff concentrator. Protein concentration was determined by the Bradford assay (35).

### Spectroscopic absorption analysis

Absorption spectra of purified Pc-Phr were recorded using a UV–visible spectrophotometer (Agilent, USA) following the method described by Dikbas et al. (7). To achieve the fully oxidized state of the cofactor, the purified Pc-Phr was treated with 20 mM potassium ferricyanide (K₃[Fe(CN)₆]) and incubated in the dark for 40 h (14). The oxidant was then removed using a Pierce protein concentrator (10K MWCO PES, Thermo Scientific). To generate a reduced cofactor state, 5 mM dithiothreitol was added to the protein solution, which was then incubated in the dark for 5 min (5). The absorption spectra were recorded in a dark-adapted state (dark state). Subsequently, the sample was exposed to continuous BL (458 nm, 120 µmol/m²·s) using a light-emitting diode (LED) system (36), and time-dependent absorption spectral changes were monitored in the range of 250–700 nm.

### *In vitro* photorepair activity assay

A single-stranded oligonucleotide, oligo(dT)16 (Bionics Co., Ltd., South Korea), was used as the DNA substrate for *in vitro* repair assays. The oligo(dT)_16_ (5′-TTT TTT TTT TTT TTT T-3′) solution was exposed to UV-C radiation (254 nm) generated using a UV hand lamp (VL-6LC, Vilber Lourmat, France) to produce CPD substrates. CPD formation was monitored by absorption spectroscopy at 260 nm at the start and hourly over a 9-h period to track the decrease in the absorbance as an indicator of CPD lesions (CPD-damaged oligo(dT)_16_) (14). To test the *in vitro* photorepairing activity of Pc-Phr, 1 μM of purified Pc-Phr was added to a repair buffer (50 mM Tris–HCl, 1 mM EDTA, 100 mM NaCl, 10% glycerol, and 1 mM dithiothreitol, pH 7.6) containing 5 μM UV-irradiated oligo(dT)_16_. The mixture was incubated in the dark for 15 min and then exposed to BL (458 nm, 50 µmol/m²·s) for 90 min. Absorption spectroscopy was used to assess the enzymatic repair activity by tracking the increase in absorption at 260 nm every 10 min following BL illumination. Bovine serum albumin (BSA) was used as the negative control.

### Removal of *phr* using CRISPR-CAS9

The *phr* gene (from start to stop codon) of PcJBC1 was knocked-out using the pCasPA/pACRISPR system by following the procedures described by Huong et al. (37) with minor modifications. The Benchling tool (https://benchling.com/) was used to design a single-guide RNA targeting *phr* (sgRNA-*phr*) to target sequences adjacent to the protospacer adjacent motif NGG. The sgRNA was generated by annealing oligonucleotides Sg_PHR_F and Sg_PHR_R (Table S1). To construct the pACRISPR-sgRNA-*phr* plasmid, the native guide RNA sequence in the pACRISPR vector was replaced with the annealed sgRNA-phr oligonucleotides using the Golden Gate Assembly, and the resulting plasmid was used for the transformation of *E. coli* TOP10. Transformants with the pACRISPR-sgRNA-*phr* plasmid were screened on ampicillin plates (100 μg/mL) and confirmed by PCR using primers Sg_PHR_F/R_Amp. Simultaneously, 500 bp upstream and downstream fragments of *phr* were amplified separately by PCR. Forward upstream (PHR_Us_F) and reverse downstream (PHR_Ds_R) primers were designed with 20–40 bp flanks homologous to the pACRISPR plasmid, whereas the reverse upstream (PHR_Us_R) and forward downstream (PHR_Ds_F) primers shared 30–40 bp overlapping sequences. Following restriction digestion of pACRISPR–sgRNA–*phr* with XbaI and XhoI, the vector was assembled with upstream and downstream fragments via the Gibson assembly to obtain the pACRISPR-sgRNA-*phr*-Us-Ds plasmid and transformed into *E. coli* TOP10. The transformants were screened on LB plates containing ampicillin (100 μg/mL) and then confirmed by PCR and sequencing using primers PHR_Us_F/PHR_Ds_R. The pACRISPR-sgRNA-*phr*-Us-Ds plasmid was electroporated into PcJBC1 cells containing the pCasPA plasmid. The electroporated cells were seeded in 1 mL LB broth medium and were incubated at 30°C for 2 h. Then, they were spread on LB agar plates containing 100 μg/mL tetracycline and 150 μg/mL ampicillin. The *phr*-deficient mutant (JBC1^Δphr^) was verified by PCR and sequenced with the PHR_Us_F/ PHR_Ds_R primers (Fig. S1A through C and S2). The plasmids were eliminated by streaking the mutants onto LB plates containing 5% (wt/vol) sucrose.

### Complementation of the knockout mutant

To complement the JBC1^Δphr^ strain, the *phr* gene and its promoter region were amplified by PCR using primers PHR_HindIII_500Us_F and PHR_BamHI_R (Table S1). The PCR amplicons were purified and cloned into the pUCP18 vector using *Hind*III/*Bam*HI restriction sites. The recombinant plasmid (*pUCP_phr*) was then transformed into *E. coli* TOP10. The transformants were screened on ampicillin plates (100 μg/mL) and confirmed through PCR and sequencing using primers M13F (−40)/M13pUC-R. The candidate gene was cloned in the orientation opposite to that of the lacZ promoter to ensure that its native promoter, rather than the lacZ promoter, drove gene expression. The constructed plasmid *pUCP*_*phr* was transformed into JBC1^Δphr^ to generate the complemented strain (JBC1^Δphr^ + pphr). In parallel, to control for potential plasmid-associated effects, the native *phr* promoter region alone was amplified using primers PHR_HindIII_500Us_F and pPHR_BamHI_R (Table S1) and cloned into pUCP18, yielding a vector control plasmid. This construct was transformed into JBC1^Δphr^ to generate the vector control strain (JBC1^Δphr^+p18), which carries the empty vector containing only the *phr* promoter and was included in all relevant experiments. Successful construction of the complemented and the vector control strains was confirmed by PCR and sequencing using the M13F(−40)/M13pUC-R primers (Fig. S1B, D and S3).

### UV-C and BL tolerance assay

Each bacterial strain (i.e., PcJBC1, JBC1^Δphr^, JBC1^Δphr^+p18, and JBC1^Δphr^+pphr) was cultured in an LB broth supplemented with vancomycin (100 μg/mL) for PcJBC1 and JBC1^Δphr^, and vancomycin (100 μg/mL) and ampicillin (100 μg/mL) for JBC1^Δphr^+p18 and JBC1^Δphr^*+*pphr at 28°C and 180 rpm in darkness. Subsequently, they were adjusted to 0.2 OD_600_ and serially diluted. LB agar plates were spread with 100 µL of approximately 10^3^ CFU/mL. The plates were exposed to UV-C irradiation using a UV hand lamp (VL-6LC, Vilber Lourmat, France; λ_max_ at 254 nm). According to the manufacturer’s specifications, the lamp emits UV-C radiation at an intensity of 400 µW/m^2^ measured at a distance of 15 cm. For the experiments, the lamp was positioned 45 cm above the plates, and the samples were exposed for 10, 20, 30, 45, 60, 90, and 120 s. The plates were then incubated at 28°C in the dark condition for 48 h. In another experiment, plates were irradiated with BL for 48 h at 28°C with intensities of 2.5, 5.0, and 7.5 µmol/m²·s using LED light sources (wavelength range from 448 to 475 nm, with a typical light emission at 458 nm), which were connected to a circuit box to control the light intensity (27). The photosynthetic photon flux density (µmol photons/m²·s) was determined using a quantum sensor (LI-190R, Li-Cor, Lincoln, USA) as necessary. The colonies were counted 48 h after incubation in the dark. Each experiment was independently repeated three times, with three replicates per treatment.

### *In vivo* photoreactivation assay

Photoreactivation assays were performed as described by Worthington et al. (3), with minor modifications. Briefly, each bacterial strain (PcJBC1, JBC1^Δphr^, JBC1^Δphr^+p18, and JBC1^Δphr^+pphr) was spread on LB agar plates, as described earlier. After exposure to UV-C (254 nm) for 45 s, the plates were exposed to BL at an intensity of 2.5 μmol/m²·s for 2 h. The plates were immediately sealed after treatment and incubated in the dark at 28°C for 48 h. The plates were incubated in the dark without any light treatment, and the plates treated with only BL were used as non-treated and BL-only controls, respectively. The survival rates were determined by calculating the ratio of CFUs in UV- and BL-treated plates to CFUs in untreated plates. Each experiment was performed thrice with three biological replicates.

### RNA isolation and RT-qPCR assay

The *phr* transcription level in PcJBC1 was assessed following the method described by He et al. (2), with minor modifications. Briefly, PcJBC1 was cultured in LB medium until the exponential growth phase was reached, and then, it was exposed to UV-C (254 nm) for 45 s or maintained in the dark condition. Subsequently, the UV-exposed cultures were immediately incubated under BL (458 nm of 2.5 µmol/m²·s) or kept in the dark for 15 min. Samples were collected by centrifugation at 14,000 rpm for 2 min at 4°C. Total RNA was isolated using RNAqueous Kit following the manufacturer’s protocol (Ambion, USA). The RNA quality and quantity were analyzed using an electropherogram (Agilent Bioanalyzer 2100). The isolated RNA was used to assess the expression level of the *phr* gene by quantitative reverse transcription PCR (RT-qPCR) using primers PHR_F/PHR_R (Table S1). The housekeeping gene *rpoD* served as an internal control. Relative expression levels were determined using the ΔΔCT method, with CT values normalized to the expression of genes in PcJBC1 grown under dark conditions. To further investigate the role of *phr* in DNA damage–associated responses, total RNA was also isolated from JBC1^Δphr^, JBC1^Δphr^+p18, and JBC1^Δphr^+pphr strains following UV-C irradiation (254 nm, 45 s) or incubation in darkness. These RNA samples were used to quantify the transcription of DNA damage–related genes, including *recA* and *uvrA*, by RT-qPCR using gene-specific primers listed in Table S1.

### Virulence assay

Each bacterial strain (PcJBC1, JBC1^Δphr^, JBC1^Δphr^+p18, and JBC1^Δphr^+pphr) was cultured overnight in LB broth at 28°C with the appropriate antibiotics. The bacterial cells were harvested by low-speed centrifugation and diluted to OD_600_ = 0.2 (1 × 10^8^ CFU/mL) in sterile 10 mM MgCl_2_ solution (27). Each bacterial strain was inoculated into a cabbage midrib following the method described by Huong et al. (37). Briefly, cabbage midribs were surface-sterilized with 70% ethanol, rinsed twice with sterile DW, and blot-dried with tissue paper. A toothpick was used to induce a 0.2 mm deep wound on the surface-disinfected midribs. The wounds were applied with 20 µL of each cell suspension. The inoculated midribs were allowed to air-dry for 10–15 min and placed on lids of Petri dishes, which were laid on two layers of paper towels (Yuhan-Kimberley, Korea) moistened with sterile DW inside plastic containers, and incubated at 25°C and >95% relative humidity in dark conditions. The diseased areas and symptoms were evaluated 3 days after incubation (DAI) by measuring the pixel areas using the Magnetic Lasso and histogram tools of Adobe Photoshop CS6 (38). At 0, 24, 48, and 72 h after inoculation, five random midrib plugs (8 mm in diameter) were taken from the inoculation areas of the kimchi cabbages treated with the bacterial suspensions. The plugs were ground in a 0.9% NaCl solution using a sterilized mortar and pestle to release the bacteria from the tissue. Serial dilutions were spread on LB agar containing vancomycin (100 mg/L). Colony counts were recorded after 48 h of incubation at 28°C and expressed as log_10_ CFU/cm². Each experiment was conducted thrice in five replicates.

### Leaf attachment assay

A leaf attachment assay was performed following the method described by Khanh and Lee (36) with minor modifications. Briefly, overnight cultures of each strain were resuspended in sterile DW at a concentration of 1 × 10^8^ CFU/mL. Whole leaves of tomato were submerged in each bacterial suspension for 2 h at 28°C in dark conditions and rinsed twice with sterile DW to remove any unattached bacteria. Six random leaf disks (8 mm in diameter) were cut and macerated in 0.9% NaCl solution using a sterilized mortar and pestle to release the attached bacteria from the leaves. The serially diluted suspensions were spread onto LB agar plates containing vancomycin (100 mg/L). Bacterial colonies were counted after 48 h of incubation at 28°C and presented on a log_10_ scale (CFU/cm^2^). The experiments were conducted in triplicate.

### H_2_O_2_ sensitivity assay

The susceptibility of the bacterial strains to H_2_O_2_ was evaluated using the disc diffusion method as described previously (39), with slight modifications. Briefly, overnight bacterial cultures were adjusted to an OD_600_ of 0.2 and incorporated into a sterilized, cooled minimal medium (MM) (40) containing 0.8% soft agar (1:20, vol/vol) before being poured into Petri dishes. A sterile 8-mm paper disk was positioned centrally, onto which 5 µL of 5% H_2_O_2_ was applied. Inhibition zone diameters were measured after 24 h of incubation at 30°C. All experiments were performed three times with three replicates.

H_2_O_2_ sensitivity was further evaluated to assess a quantitative oxidative stress tolerance using a liquid growth assay, as described previously (41, 42), with minor modifications. Overnight cultures were harvested and resuspended in fresh LB. medium to an initial density of approximately 1 × 10^7^ CFU/mL. Aliquots of 100 µL of the bacterial suspension were dispensed into wells of a 96-well microtiter plate containing 100 µL of LB medium supplemented with H₂O₂ at a 2× concentration, resulting in a final H_2_O_2_ concentration of 100 µM. This concentration was selected to impose sublethal oxidative stress without fully inhibiting bacterial growth. Control wells received LB medium without H_2_O_2_, and blank wells contained LB medium only. Plates were incubated at 30°C with shaking at 120 rpm, and bacterial growth was monitored by measuring OD_600_ at 1 h intervals for up to 28 h using a Varioskan^TM^ LUX multimode microplate reader (Thermo Scientific, USA). Relative survival was calculated at 16 h by normalizing OD₆₀₀ values to untreated controls using the following equation (43): percent survival = (OD_with H2O2_ – OD_blank_)/(OD_without H2O2_ – OD_blank control_) ×100. All treatments were performed in triplicate, with independent biological replicates.

### Intracellular reactive oxygen species (ROS) assay

Intracellular ROS levels were quantified using the fluorescent probe 2′,7′-dichlorodihydrofluorescein diacetate (DCFH-DA; Sigma-Aldrich, USA) following Castro-Alférez et al. (44) with minor modifications. For oxidative stress assays, bacterial strains (PcJBC1, JBC1^Δphr^, JBC1^Δphr^+p18, and JBC1^Δphr^+pphr) were grown in minimal medium (40) at 30°C with shaking at 180 rpm to the exponential phase and adjusted to an equal cell density (10^8^ CFU/mL) and treated with 6 mM H_2_O_2_ for 2 h at 30°C. For UV stress assays, bacterial cultures grown in LB to the exponential phase were adjusted to the same cell density and placed in open Petri dishes (6 mm in diameter). The cultures were exposed to UV-C irradiation (254 nm) using a UV hand lamp (VL-6LC, Vilber Lourmat, France; λ_max_ at 254 nm, 400 µW/m^2^) for 2 min at a distance of 45 cm. Untreated cultures maintained in darkness served as controls. Following treatments, cells were harvested by centrifugation (5,000 rpm, 5 min), washed twice with phosphate-buffered saline (PBS, pH 7.2), and resuspended in PBS. DCFH-DA was dissolved in dimethyl sulfoxide and added to a final concentration of 10 μM, and samples were incubated in the dark at 28°C for 45 min. Excess dye was removed by washing with PBS. Fluorescence intensity was measured using a microplate reader at excitation 488 nm and emission 525 nm (44). Fluorescence values were normalized to OD_600_ to account for variations in cell density. The fold change in ROS levels was then calculated as the ratio of normalized ROS in treated samples to that in control samples. All experiments were performed with three independent biological replicates, each analyzed in technical triplicate.

### Statistical analysis

Data were analyzed using Minitab software version 16.2.0 (45). Significant differences among multiple treatment groups were determined using one-way analysis of variance (ANOVA) followed by Tukey’s post-hoc test. For comparisons between two specific groups, Student’s *t*-test was employed. Statistical significance was defined as *P* ≤ 0.05, with specific levels indicated by asterisks, *P ≤ 0.05, **P ≤ 0.01, ***P ≤ 0.001.

## RESULTS

### Domain architecture and phylogenetic classification of Pc-Phr

**Fig 1:**
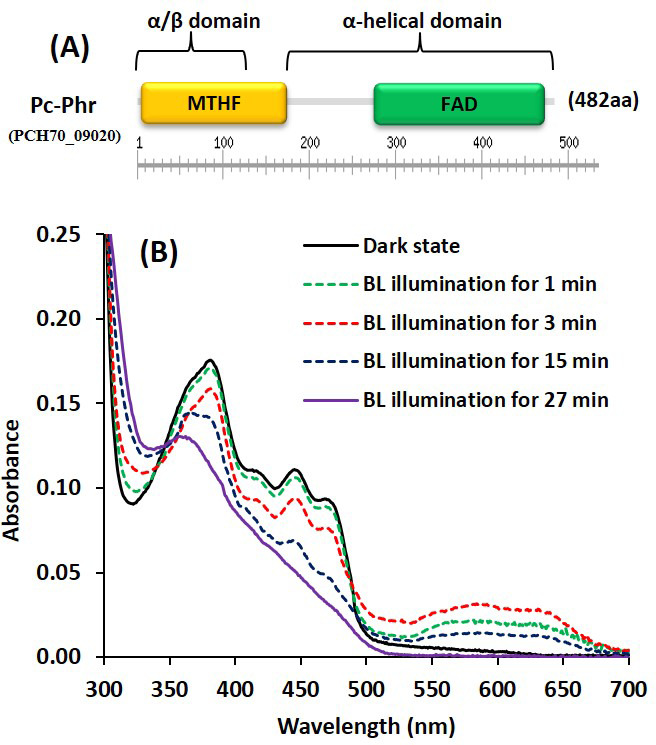
Domain structure and photochemical properties of Pc-Phr. (**A**) Schematic of the domain architecture of the Pc-Phr protein from *P. cichorii* JBC1. The NCBI gene locus tag is included in parentheses. The diagram illustrates the full-length protein, the predicted domain types, and their relative positions. Pc-Phr contains an α/β domain with a secondary pocket (yellow) capable of binding to a secondary antenna chromophore such as MTHF and an α-helical domain that contains a FAD-binding pocket (green). (**B**) Absorption spectral changes of Pc-Phr under BL exposure, showing the transition from the oxidized flavin state (FADox, Dark state) to the semiquinone radical (FADH•) and subsequently to the fully reduced form (FADH^−^), as indicated by characteristic absorption maxima. Spectral measurements were conducted at 25°C in the presence of dithiothreitol.

Genome analysis of PcJBC1 using the CDD tool identified a gene (NCBI locus tag PCH70_09020) that encodes a putative Phr, which is designated as Pc-Phr. Pc-Phr consists of 482 amino acids, with a predicted MW of approximately 54.5 kDa. Domain analysis revealed a typical photolyase architecture, consisting of an N-terminal DNA photolyase domain predicted to bind a light-harvesting antenna chromophore and a C-terminal domain responsible for binding the catalytic FAD cofactor (Fig. 1A). Phylogenetic analysis based on amino acid sequence alignment with representative members of the CPF showed that Pc-Phr belongs to class I CPD Phrs (Fig. S4). Multiple sequence alignment revealed that Pc-Phr shares 79.3%, 44.6%, and 41.5% identity with Phrs from *P. koreensis*, *E. coli* K-12, and *V. cholerae* O1, respectively (Fig. S5; Table S2). Similar to other class I CPD Phrs, Pc-Phr contains a highly conserved tryptophan triad (W385–W362–W309) that is essential for photoinduced electron transport during DNA repair. This triad is homologous to the canonical W382–W359–W306 motif found in *E. coli* Phr (46) (Fig. S5). These sequence and structural features support the classification of Pc-Phr as a functional class I CPD photolyase.

### UV–Visible absorption properties of Pc-Phr

The UV–visible spectral characteristics of Pc-Phr were analyzed following its overexpression and purification from *E. coli*. Figure 1B shows that under dark conditions, Pc-Phr exhibits two prominent absorbance peaks. One peak is observed at 380 nm, corresponding to the light-harvesting antenna cofactor 5,10-MTHF. The other peak is at 445 nm, which is characteristic of oxidized FAD (FADox) bound to the Phr catalytic site. These spectral features are consistent with those observed in *E. coli* and other characterized Phrs (5, 47, 48). Upon BL exposure, Pc-Phr undergoes a photoreduction reaction that converts its catalytic FAD into its catalytically active, fully reduced form, FADH^−^. An initial intermediate semiquinone radical state (FADH^●^) is evident from the transient emergence of absorption peaks at 587 and 632 nm, which is consistent with previous reports (49). Prolonged BL exposure results in the disappearance of these peaks and a concomitant increase in the 360 nm peak, as shown in Fig. 1B, which indicates the full reduction of FADox to FADH^−^ via intraprotein electron transfer (49, 50). These results indicate that Pc-Phr contains canonical Phr cofactors and undergoes light-driven redox transitions required for CPD repair.

### *In vitro* photorepair activity of Pc-Phr

**Fig 2:**
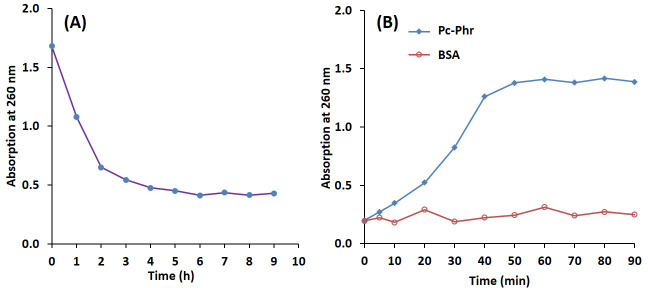
Photorepair activity of purified Pc-Phr on UV-induced DNA damage under blue light exposure. (**A**) DNA damage was assessed by measuring the absorbance at 260 nm of oligo (dT)_16_ following UV-C irradiation. (**B**) The solution containing UV-C-irradiated oligo (dT)_16_ was incubated with purified Pc-Phr under BL (50 µmol/m²·s). Subsequently, the absorbance at 260 nm was monitored over time to evaluate the Pc-Phr-mediated photorepair activity. BSA was used as the negative control.

Phr proteins repair UV-induced DNA damage by absorbing photons within the BL and UV-A regions (300–500 nm). To evaluate the catalytic activity of the purified Pc-Phr expressed in *E. coli* BL21, we conducted an *in vitro* photorepair assay using UV-C-damaged oligo(dT)_16_. UV-C (254 nm) irradiation significantly reduced the absorbance at 260 nm from 1.7 to 0.4, which indicated CPD lesion formation in oligo(dT)_16_ (Fig. 2A). Upon incubation with purified Pc-Phr followed by BL illumination, the absorbance at 260 nm increased gradually, reaching 1.26 after 40 min, consistent with CPD repair and restoration of normal base-stacking arrangement. In contrast, no significant recovery in absorbance was observed in control reactions containing BSA instead of Pc-Phr (Fig. 2B). These results confirm that Pc-Phr possesses intrinsic CPD photorepair activity and repairs UV-induced DNA lesions in a light-dependent manner.

### Impact of Pc-Phr on UV-C and BL sensitivity in Pc-JBC1

To determine the contribution of Pc-Phr to UV tolerance, PcJBC1, JBC1^Δphr^, JBC1^Δphr^+p18, and JBC1^Δphr^+pphr were exposed to UV-C (254 nm) for varying durations (10, 20, 30, 45, 60, 90, and 120 s), and their survival rates were assessed. JBC1^Δphr^ and JBC1^Δphr^+p18 exhibited significantly reduced survivability under prolonged UV-C exposure compared to PcJBC1 and JBC1^Δphr^+pphr. UV-C treatment for 10 s decreased the PcJBC1 survival rate by only 4% compared to the dark control, whereas exposure for 30 s reduced its viability by more than 60%. UV-C treatment for 20 s significantly decreased the survival rate of JBC1^Δphr^ (63.4%) compared to PcJBC1 (82.2%). The complemented strain showed enhanced survivability, surpassing that of the wild-type strain under UV-C conditions (Table 1). These observations suggest that Pc-Phr is important for mitigating UV-C-induced cellular damage.

**TABLE 1:** Survival of *Pseudomonas cichorii* and its mutant strains after treatment with UV-C radiation (254 nm)^*a*^

Strain	Survival rate (%) after each exposure duration							Dark (control)
	10 s	20 s	30 s	45 s	60 s	90 s	120 s	
PcJBC1	96.0 ± 0.8^a^	82.2 ± 0.5^c^	38.2 ± 2.1^ef^	31.8 ± 2.7^g^	11.9 ± 2.4^j^	1.6 ± 0.2^n^	0^o^	100^p^
JBC1^Δphr^	94.9 ± 2.7^a^	63.4 ± 3.2^d^	33.7 ± 5.0^f^	24.7 ± 2.2^h^	7.2 ± 0.5^k^	1.2 ± 0.1^n^	0^o^	100^p^
JBC1^Δphr^+p18	94.2 ± 1.9^a^	60.5 ± 2.7^d^	32.0 ± 3.9^f^	23.5 ± 1.6^h^	6.8 ± 1.5^k^	1.2 ± 0.2^n^	0^o^	100^p^
JBC1^Δphr^+pphr	95.5 ± 0.8^a^	84.2 ± 1.9^c^	46.5 ± 2.7^e^	35.8 ± 2.4^g^	17.8 ± 1.6^j^	3.6 ± 0.7^m^	0^o^	100^p^

^*a*^ Bacterial suspensions were spread on LB plates and exposed to UV-C (254 nm) for 10, 20, 30, 45, 60, 90, and 120 s and then immediately incubated in the dark at 28℃ for 48 h. Values represent the mean ± standard deviation (SD) of three biologically independent experiments performed in triplicate. In the same column, means with identical letters indicate no significant difference (*P* > 0.05, Tukey’s test).

**Fig 3:**
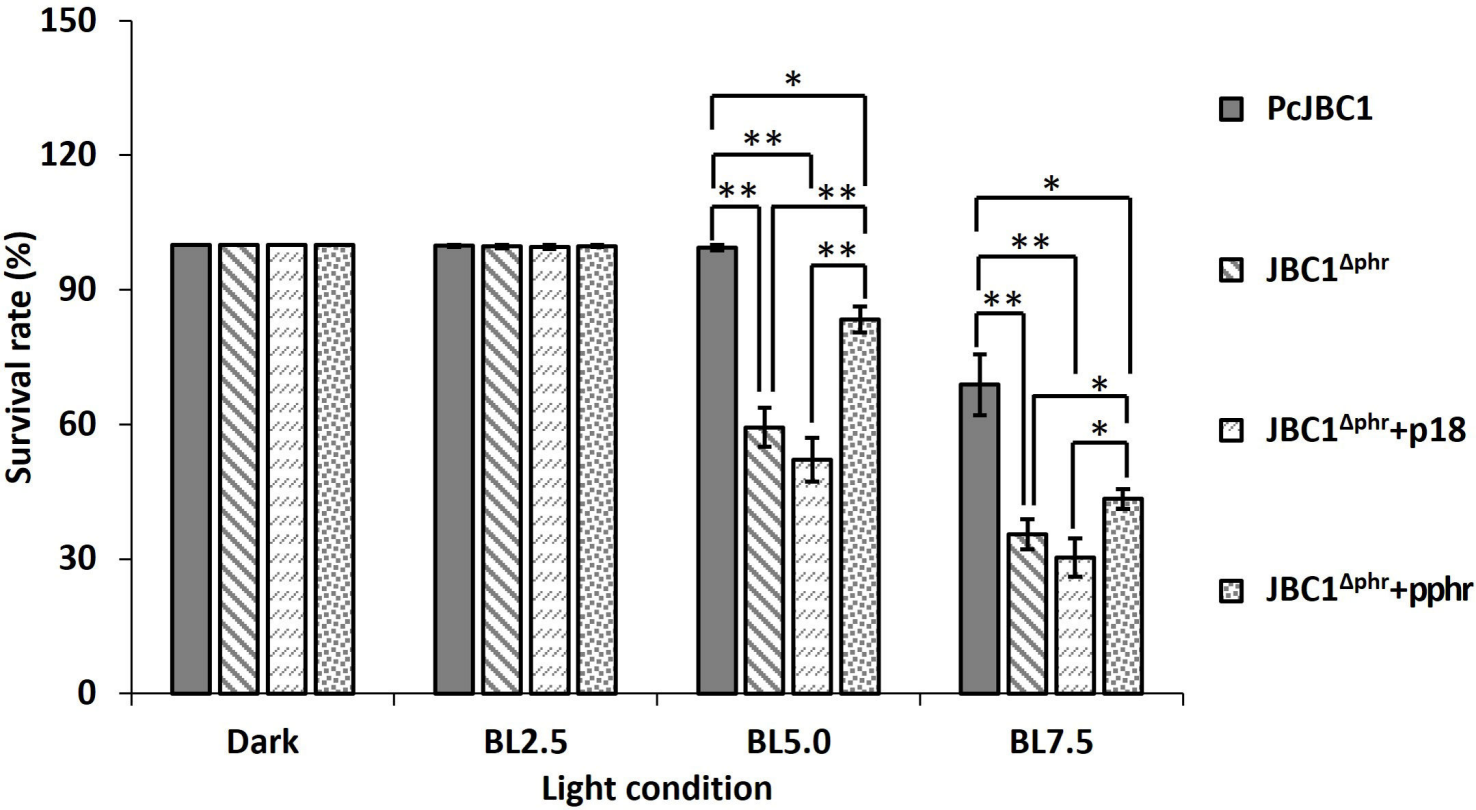
Effect of Pc-Phr and blue light on survival of *P. cichorii* JBC1. Bacterial cells were spread on LB plates and incubated at 28°C under BL at intensities of 2.5, 5, and 7.5 µmol/m²·s and in dark conditions for 48 h. Bars represent means ± SD from three independent experiments, each performed in triplicate, and *P* values are indicated by asterisks, *P ≤ 0.05, **P ≤ 0.01, ***P ≤ 0.001, according to Student’s *t*-test.

In addition, the strains were exposed to different BL intensities (2.5, 5.0, and 7.5 µmol/m²·s) for 48 h to assess the BL sensitivity. At 2.5 µmol/m²·s, all strains maintained a survival rate comparable to that under dark control. However, as the BL intensity increased, the survival of JBC1^Δphr^ and JBC1^Δphr^+p18 significantly decreased to 59.4% and 52.1% at 5 µmol/m²·s, whereas PcJBC1 maintained a high viability (99.4%). At 7.5 µmol/m²·s, JBC1^Δphr^ and JBC1^Δphr^+p18 survival further decreased to 34.6% and 30.4%, respectively, whereas the complemented strain showed intermediate survival (83.3% and 43.4% at 5.0 and 7.5 µmol/m²·s, respectively), although not fully restored to wild-type levels (99.4% and 68.9%, respectively) (Fig. 3; Table S3). Overall, these results indicate that Pc-Phr significantly enhances the resistance of PcJBC1 to UV-C and high-intensity BL damage, and the empty vector pUCP18 did not contribute to UV-C and BL tolerances. Based on these results, a sub-lethal BL intensity of 2.5 µmol/m²·s was selected for the subsequent experiments.

### Photoreactivation of UV-C-induced DNA damage

**Fig 4:**
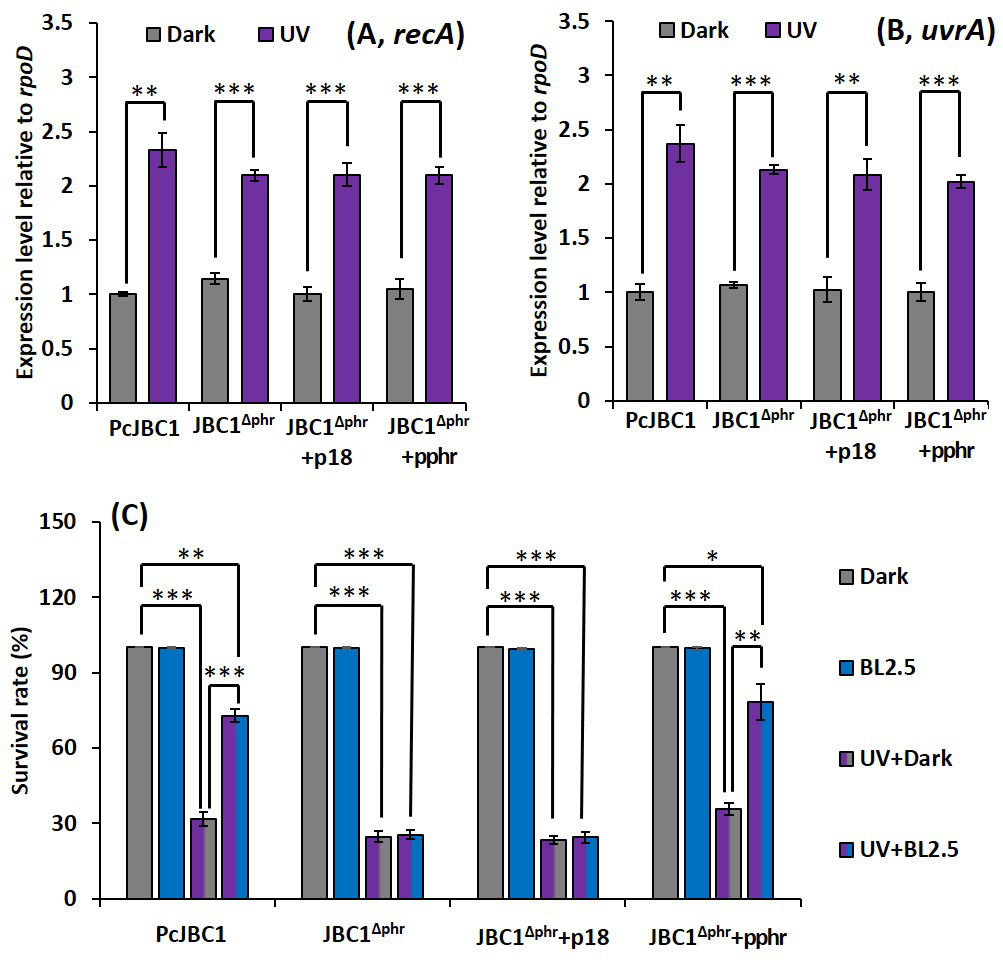
DNA damage response under UV radiation and the role of the *phr* gene in the photoreactivation of *P. cichorii* JBC1. Relative expression of the DNA damage response genes (**A**) *recA* and (**B**) *uvrA* following UV-C exposure. Bacterial strains (PcJBC1, JBC1^Δphr^, JBC1^Δphr^+p18, and JBC1^Δphr^+pphr) were grown in LB agar under dark conditions to the exponential growth phase and either exposed to UV-C (254 nm; UV) for 45 s or maintained in darkness (Dark). The gene expression was analyzed via RT-qPCR using *rpoD* as an internal control. Relative fold-change was calculated using the ΔΔCT method, normalized to the expression level in darkness of the PcJBC1 strain. (**C**) Survival rates following photoreactivation. Cells were initially exposed to UV-C for 45 s, subsequently incubated either in complete darkness or under BL (2.5 µmol/m²·s) for 2 h to induce photoreactivation, and followed by incubation in darkness. Survival rates were determined by calculating the ratio of colony numbers to the non-treated control. Bars represent the means ± standard deviations, and *P* values are indicated by asterisks, *P ≤ 0.05, **P ≤ 0.01, ***P ≤ 0.001, according to Student’s *t*-test.

Exposure of all tested strains (PcJBC1, JBC1^Δphr^, JBC1^Δphr^+p18, and JBC1^Δphr^+pphr) to UV-C irradiation resulted in significant induction of *recA* and *uvrA* expression (Fig. 4A and B), indicating the formation of DNA lesions and activation of the DNA damage response. The *recA* gene encodes the central regulator of the bacterial SOS response, while *uvrA* is a key component of the nucleotide excision repair (NER) pathway, which removes bulky DNA lesions. The coordinated upregulation of these genes confirms that the applied UV-C treatment was sufficient to trigger substantial DNA damage across all strains.

To evaluate the role of Pc-Phr in the photorecovery of bacterial cells from UV-induced DNA damage, bacterial strains were exposed to UV-C irradiation for 45 s followed by BL treatment (2.5 µmol/m²·s) for 2 h. UV-C exposure significantly reduced the survival rates of all strains, with PcJBC1, JBC1^Δphr^, JBC1^Δphr^+p18, and JBC1^Δphr+pphr^ exhibiting survival rates of 31.8%, 24.7%, 23.5%, and 35.8%, respectively (Fig. 4C). Under subsequent BL treatment, PcJBC1 and the complemented strain showed marked recovery, with survival rates increasing to 72.8% and 78.2%, respectively, whereas JBC1^Δphr^ and JBC1^Δphr^+p18 did not exhibit significant recovery (25.6% and 24.4%, respectively). These results indicate that Pc-Phr plays a key role in repairing UV-induced damage in PcJBC1 and that its DNA repair activity is specifically activated by BL.

### Expression of the *phr* gene in PcJBC1

**Fig 5:**
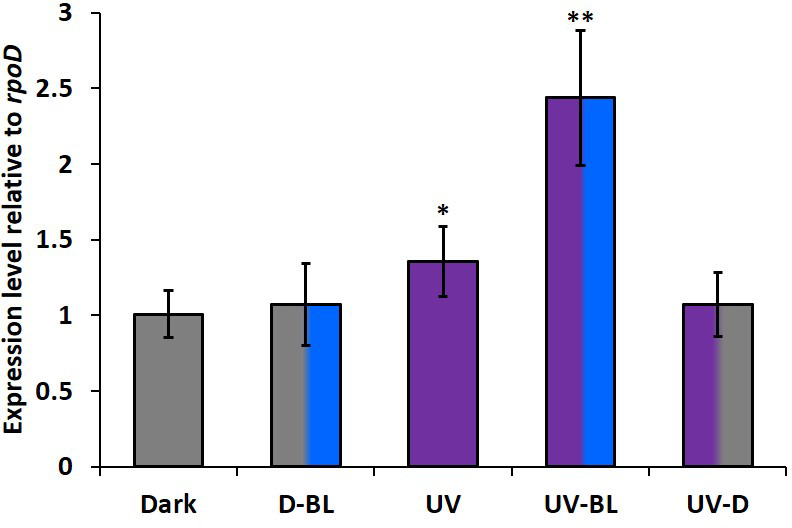
Effects of blue light and UV-C on *phr* expression in *P. cichorii* JBC1. The pathogen was cultured in LB agar under dark conditions until the exponential growth phase was reached. Cells were then exposed to UV-C (254 nm) for 45 s or maintained in darkness (Dark). Following treatment, the cultures were incubated for 15 min either under BL (458 nm at 2.5 µmol/m²·s; UV-BL and D-BL, respectively) or in the dark condition (UV-D). The total RNA was extracted, and the *phr* expression was analyzed by RT-qPCR using *rpoD* as the internal control. Relative gene expression was determined using the ΔΔCT method, normalized to the expression level in darkness. Bars represent the mean ± SD of five independent biological replicates. Statistical significance between treatment groups and Dark control was determined by Student’s *t*-test; asterisks indicate significance levels: *P ≤ 0.05, **P ≤ 0.01, ***P ≤ 0.001.

To understand *phr* gene expression in PcJBC1 in response to light stimuli, we analyzed *phr* gene expression under UV-C and BL exposure. Basal *phr* expression was low under dark conditions. However, UV-C irradiation for 45 s induced *phr* expression (1.4-fold), and subsequent exposure to BL for 15 min significantly upregulated *phr* expression by 2.4-fold (Fig. 5; Fig. S6; Table S4). In contrast, transfer of UV-C-treated cells to the dark for 15 min resulted in decreased *phr* expression, similar to that in cells maintained under continuous darkness. Furthermore, BL exposure alone (15 min), without prior UV-C irradiation, did not significantly alter the *phr* expression. These results indicate that *phr* expression is primarily induced by UV-C damage and further enhanced by BL, supporting coordinated light-dependent regulation.

### Effects of Pc-Phr on virulence and *in planta* survival of PcJBC1

**Fig 6:**
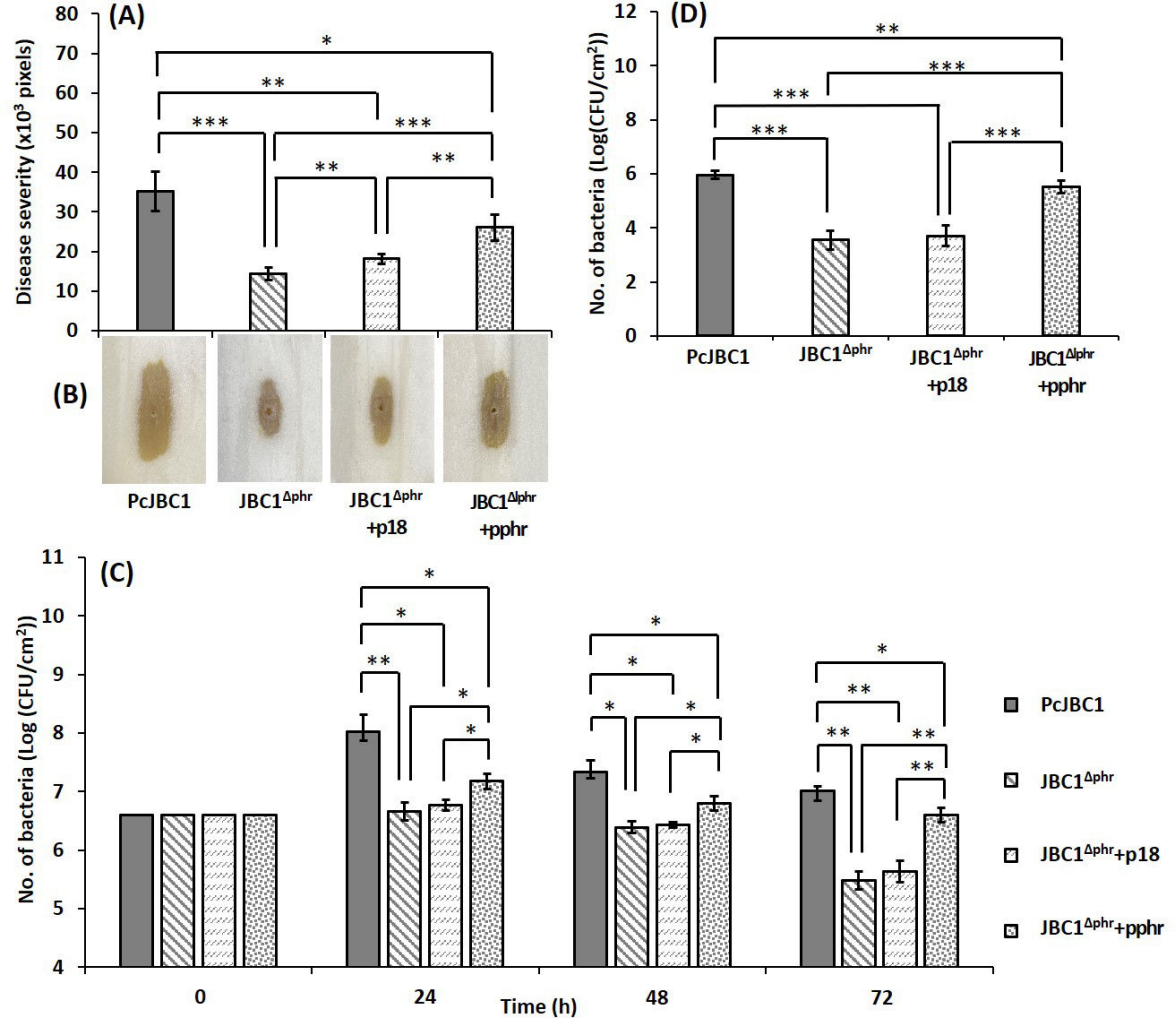
Effects of Pc-Phr on the virulence, bacterial fitness, and epiphytic attachment of *P. cichorii* JBC1. (**A**) Wounded cabbage midribs were inoculated with 20 μL of bacterial suspension (1 × 10^8^ CFU/mL) of PcJBC1, JBC1^Δphr^, JBC1^Δphr^+p18, and JBC1^Δphr^+pphr and incubated at 25°C in the dark with 90% relative humidity. Disease severity was quantified using Adobe Photoshop CS6. (**B**) Representative disease symptoms observed on cabbage midribs three days after inoculation. (**C**) Bacterial populations were quantified from the inoculation sites 0, 24, 48, and 72 h after inoculation. (**D**) Tomato leaves are submerged for 2 h in bacterial suspensions (1 × 10^8^ CFU/mL) at 28°C under dark conditions. The population of epiphytically attached bacterial cells was retrieved, plated on LB agar plates, and quantified by colony counting. Bars represent the means ± SD of five independent biological replicates, and *P* values were indicated by asterisks, *P ≤ 0.05, **P ≤ 0.01, ***P ≤ 0.001, according to Student’s *t*-test.

To investigate the role of Pc-Phr in the virulence of *P. cichorii*, we compared the virulence of the *phr* deletion mutants (JBC1^Δphr^ and JBC1^Δphr^+p18) with that of the wild-type (PcJBC1) and complemented strains (JBC1^Δphr^+pphr) by inoculating cabbage midribs (Fig. 6A and B). The disease severity caused by JBC1^Δphr^ (14.5 × 10^3^ pixels) was significantly lower than that by PcJBC1 (35.1 × 10^3^ pixels). The mutant harboring the empty pUCP18 vector (JBC1^Δphr^+p18) exhibited slightly higher virulence (18.2 × 10^3^ pixels) compared to JBC1^Δphr^; however, it was significantly lower than the complemented strains (26.0 × 10^3^ pixels). The supplemented strain showed partial restoration of virulence. To further investigate the contribution of Pc-Phr to bacterial fitness *in planta*, we monitored bacterial population dynamics in cabbage midrib tissues (Fig. 6C). Although all strains showed initial growth 24 h post-inoculation (hpi), the population steadily declined. The loss of the *phr* gene significantly decreased the bacterial counts at 24, 48, and 72 hpi in JBC1^Δphr^ (log CFU/cm^2^ of 6.7, 6.4, and 5.5, respectively) and in JBC1^Δphr^+p18 (log CFU/cm^2^ of 6.8, 6.4, and 5.6, respectively) compared to the wild-type strain (8.0, 7.3, and 7.0, respectively). The complemented strain showed intermediate population levels (7.2, 6.8, and 6.6, respectively), thereby indicating incomplete restoration. These results suggest that Pc-Phr contributes to *in planta* survival and growth of PcJBC1.

### Leaf attachment

Bacterial adherence to plant surfaces is an initial step in the infection process, as it enables survival and subsequent colonization. In this study, the attachment of JBC1^Δphr^ and JBC1^Δphr^+p18 on a tomato leaf surface was significantly reduced (3.5 and 3.7 log CFU/cm^2^, respectively) compared to that of the wild-type strain (6.0 log CFU/cm^2^) (Fig. 6D). Complementation of Pc-Phr into JBC1^Δphr^ partially restored adhesion (5.5 log CFU/cm^2^); however, it was lower than that of the wild-type. These results indicate that Pc-Phr contributes to efficient bacterial attachment during early stages of host colonization.

### H_2_O_2_ sensitivity

**Fig 7:**
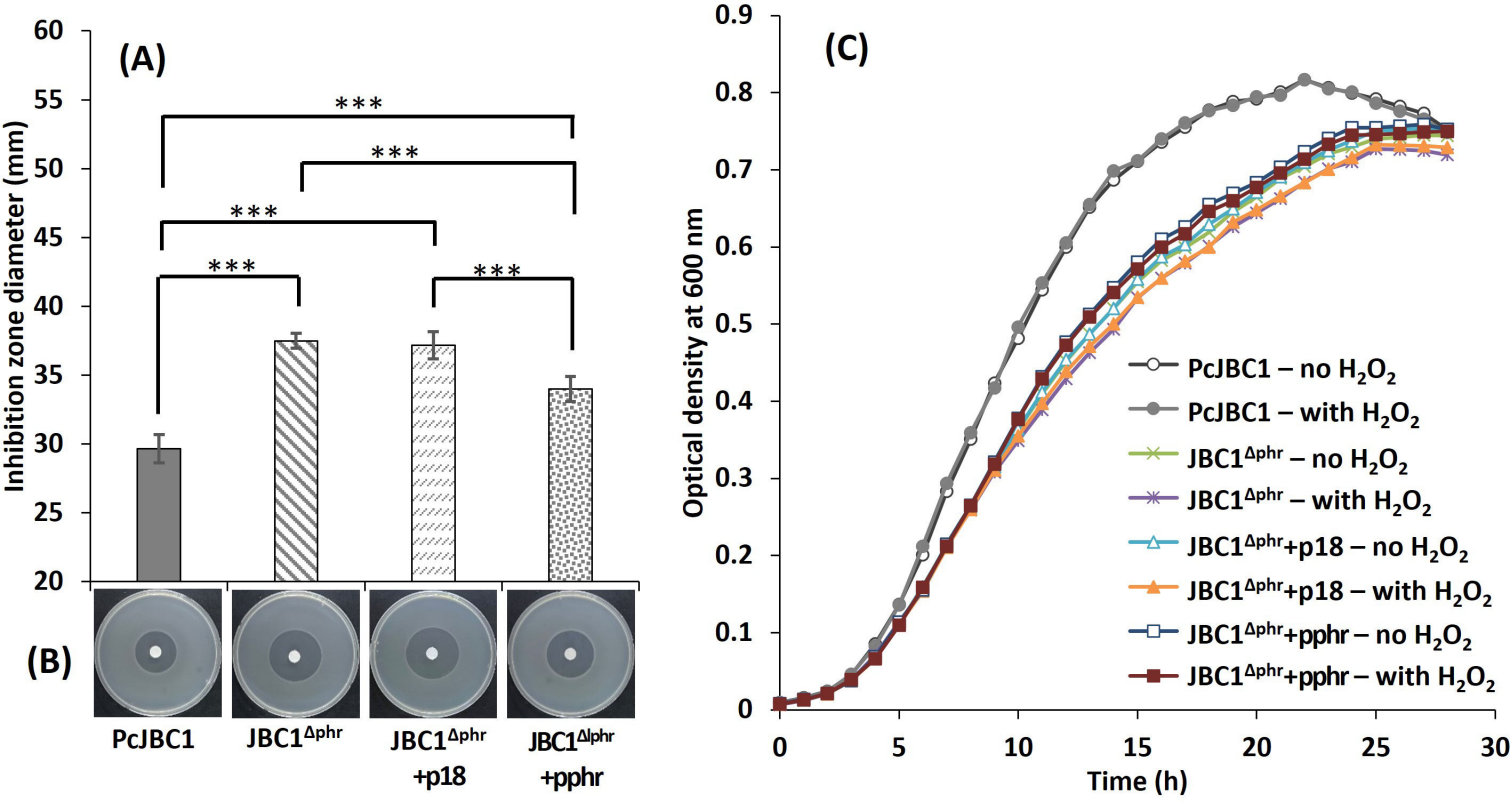
Effects of Pc-Phr on hydrogen peroxide sensitivity in *P. cichorii* JBC1. (**A**) Sensitivity to oxidative stress was assessed by measuring the diameters of the growth inhibition zones following exposure to H_2_O_2_ after 24 h of incubation at 30°C. Bars represent the means ± SD from three independent experiments, and *P* values are indicated by asterisks, *P ≤ 0.05, **P ≤ 0.01, ***P ≤ 0.001, according to Student’s *t*-test. (**B**) Representative images of an inhibition zone taken 24 h after incubation. (**C**) Growth kinetics of bacterial strains in LB medium in the presence of H_2_O_2_ (100 µM; with H_2_O_2_), or in the absence of H_2_O_2_ (no H_2_O_2_), monitored for 28 h at 30°C with shaking at 120 rpm.

During epiphytic growth and host tissue colonization, plant-pathogenic bacteria are exposed to various stressors, including H_2_O_2_, which serves as both a signaling molecule in plant defense and a toxic byproduct of metabolism. JBC1^Δphr^ and JBC1^Δphr^+p18 mutant strains exhibited a marked decline in the bacterial population on cabbage midribs; therefore, we examined the contribution of Pc-Phr to oxidative stress resistance by measuring the inhibition zones in response to H_2_O_2_. Both mutant strains showed a significantly increased sensitivity to H_2_O_2_ oxidative stress, with a mean inhibition zone of 37.5 mm and 37.1 mm, respectively, compared to PcJBC1 (29.7 mm) and JBC1^Δphr^+pphr (34.0 mm) (Fig. 7A and B).

Under sublethal H_2_O_2_ (100 µM), the growth kinetics of two mutant strains significantly decreased compared to their respective controls grown in the absence of H_2_O_2_. The complemented strain showed a slight reduction in growth, whereas the growth of the wild-type strain was not noticeably affected by this H_2_O_2_ concentration (Fig. 7C). At 16 h, the percent survival (relative growth) of PcJBC1 and JBC1^Δphr^+pphr was 100% and 98.3%, respectively, which was higher than that observed for JBC1^Δphr^ and JBC1^Δphr^+p18 (95.1% and 95.2%, respectively). These results indicate that Pc-Phr enhances resistance to oxidative stress, which probably contributes to survival and virulence under host-induced ROS conditions.

### Pc-Phr contributes to intracellular ROS homeostasis under UV and oxidative stress

**Fig 8:**
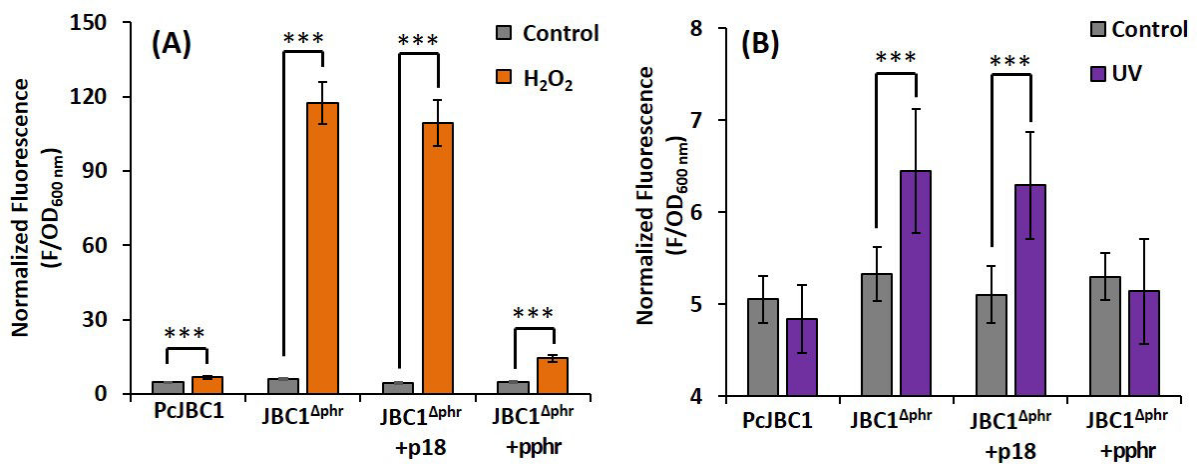
Intracellular ROS levels in *P. cichorii* JBC1 and derivative strains under oxidative and UV stress. Intracellular ROS levels were measured using the DCFH-DA fluorescence assay. Bacterial strains were subjected to (**A**) H_2_O_2_ treatment (6 mM, 2 h) and (**B**) UV-C irradiation (254 nm, 2 min). Untreated cultures maintained in the dark served as controls. Fluorescence intensity was normalized to OD_600 nm_. Data represent the mean ± standard deviation of three independent biological replicates. Statistical significance between treatment groups and dark control was determined by Student’s *t*-test, and asterisks indicate significance levels: *P ≤ 0.05, **P ≤ 0.01, ***P ≤ 0.001.

To determine whether Pc-Phr influences intracellular redox balance, ROS levels were quantified under both baseline and stress conditions. Under non-stress conditions, all strains exhibited comparable basal ROS levels (Fig. 8), indicating that deletion of *phr* does not disrupt redox homeostasis in the absence of external stressors. Exposure to 6 mM H_2_O_2_ resulted in a substantial increase in ROS levels across all strains. However, the JBC1^Δphr^ and JBC1^Δphr^+p18 mutants exhibited markedly higher ROS accumulation, with 18.9-fold and 24.8-fold increases relative to their respective controls. In contrast, the wild-type PcJBC1 and the complemented strain JBC1^Δphr^+pphr maintained significantly lower ROS levels, showing only 1.4-fold and 3.0-fold increases, respectively (Fig. 8A). Similarly, UV-C irradiation induced ROS generation in bacterial strains. The *phr*-deficient mutant and vector control displayed significantly elevated normalized fluorescence intensities (6.5 and 6.3, respectively), whereas the wild-type and complemented strains effectively mitigated ROS accumulation, maintaining levels comparable to dark controls (Fig. 8B). These results demonstrate that Pc-Phr plays a crucial role in mitigating oxidative damage and maintaining intracellular redox homeostasis following UV and H₂O₂ exposure.

## DISCUSSION

Solar radiation is essential for life; however, its UV component represents a potent genotoxic threat, primarily through the induction of DNA lesions such as CPDs and (6–4)PPs (4–6). To mitigate this damage, many organisms rely on photolyases (Phrs)—a highly conserved subclass of flavoproteins within the CPF—that catalyze the light-dependent repair of UV-induced DNA lesions. In this study, we identified and characterized Pc-Phr*,* a class I CPD photolyase from PcJBC1, and demonstrated its integral role in photoreactivation, stress tolerance, and pathogenicity.

Sequence and structural analyses revealed that Pc-Phr possesses a canonical architecture of class I CPD photolyases, including a conserved bilobal configuration with a C-terminal FAD-binding domain essential for catalytic activity, and an N-terminal domain accommodating an antenna chromophore, typically MTHF or 8-HDF (6, 15). The antenna chromophore enhances photorepair efficiency by capturing photons in the blue-light spectrum and transferring excitation energy to FAD, which subsequently mediates CPD cleavage (8). Photoreactivation is initiated when light absorption (300–500 nm) drives the formation of the excited FADH⁻ state, enabling electron transfer to CPD lesions, cleavage of the cyclobutane ring, and restoration of DNA integrity (51, 52).

Upon blue light illumination, Pc-Phr undergoes a series of redox transitions characteristic of active photolyases, shifting from the oxidized state (FADox, 445 nm) through the semiquinone radical (FADH•, 587 and 632 nm) to the fully reduced catalytic form (FADH⁻, 360 nm), consistent with photoreduction patterns observed in other bacterial Phrs (49, 50). The presence of a conserved tryptophan triad (W385–W362–W309), analogous to that described in *E. coli* photolyase (W382–W359–W306), further supports efficient intramolecular electron transfer during photorepair (46, 49, 51). Together, these features provide strong evidence that Pc-Phr is a functionally active class I CPD photolyase capable of mediating light-dependent DNA repair in *P. cichorii*.

The bacterial SOS response is a central model for understanding DNA damage–induced stress responses in prokaryotes (53). In the present study, the strong induction of *recA* and *uvrA* following UV-C irradiation across all strains confirms that the applied UV treatment generated substantial DNA lesions, triggering activation of both the SOS response and nucleotide excision repair (NER) pathways (54–57). RecA functions as a primary sensor of DNA damage by binding to single-stranded DNA regions generated at stalled replication forks, thereby promoting LexA autocleavage and derepression of SOS-regulated genes (55). The concomitant induction of *uvrA* reflects activation of NER, a light-independent (“dark repair”) mechanism capable of removing bulky lesions such as cyclobutane pyrimidine dimers (CPDs) (56, 57).

Despite NER activation in all strains, survival analyses revealed that dark repair alone was insufficient to ensure efficient recovery following UV-C exposure. Only the wild-type PcJBC1 and the complemented strain exhibited robust recovery under blue light, whereas the *phr*-deficient mutant and vector control strains failed to regain viability. These results indicate the dominant role of Pc-Phr–mediated photoreactivation in restoring bacterial survival following UV stress. Photolyases directly reverse CPDs in a single catalytic step without the need for DNA excision or resynthesis, making them substantially more efficient than NER under photic conditions (6).

Transcriptional analyses further support this functional distinction. While SOS and NER genes were induced uniformly following UV exposure, *phr* expression was specifically enhanced by the combination of UV damage and blue light exposure, but not by blue light alone. This pattern suggests that Pc-Phr is not a constitutive light-responsive enzyme, but rather is regulated in a damage-dependent, light-activated manner. Similar regulatory features have been reported for photolyase genes in *V. cholerae* (23), *Bipolaris oryzae* (58), and *Rhodococcus* sp. NJ-530 (2), indicating that coordinated integration of DNA damage sensing and photic cues may be a conserved strategy among diverse microorganisms.

Consistent with these *in vivo* observations, Pc-Phr exhibited robust *in vitro* CPD repair activity, restoring UV-damaged oligo(dT)_16_ substrates in a time-dependent manner under blue light, as reflected by increased absorbance at 260 nm—a hallmark of CPD photoreversal (59). Together, these results position Pc-Phr as a key component of the DNA damage response network in *P. cichorii*, functioning downstream of damage sensing to promote rapid and efficient lesion repair under photic conditions.

Beyond its canonical role in DNA repair, a major finding of this study is the involvement of Pc-Phr in maintaining intracellular ROS homeostasis. Deletion of *phr* resulted in significantly elevated ROS accumulation under both H₂O₂ treatment and UV-C irradiation, indicating that Pc-Phr contributes to oxidative stress tolerance. ROS are unavoidable byproducts of aerobic metabolism and environmental stress, including UV exposure, and can damage DNA, proteins, and lipids if not properly controlled (60). UV-induced ROS production has been shown to contribute substantially to cellular damage, acting synergistically with direct DNA photolesions (60).

The elevated ROS levels observed in the *phr*-deficient mutant and vector control strains suggest that Pc-Phr indirectly contributes to oxidative stress mitigation as a second shield beyond DNA repair. This enhanced oxidative stress tolerance likely arises from the prevention of UV-induced DNA lesions, thereby limiting secondary oxidative damage and genomic instability that can otherwise exacerbate ROS production (61). Additionally, the fully reduced FADH⁻ state of photolyases has been proposed to contribute to cellular redox balance, potentially conferring ROS-protective effects. Similar multifunctional roles have been reported for cryptochrome-like photolyases in *Aspergillus nidulans*, where CryA acts as a sensor of oxidative stress and shuttles between cellular compartments to mitigate damage (62). ROS tolerance is a key determinant of pathogenic fitness in pseudomonads, enabling resistance to host-derived oxidative stress, antimicrobial compounds, and microbial antibiotics encountered in both plant tissues and environmental niches (25).

ROS also play a central role in plant–microbe interactions, functioning as both antimicrobial agents and signaling molecules (63). During infection, plants rapidly activate defense responses, most notably an oxidative burst characterized by the accumulation of ROS such as H_2_O_2_ and superoxide radicals, which function both as antimicrobial agents and as signaling molecules that activate downstream defense responses (25, 64–68). The ability of *P. cichorii* to tolerate oxidative stress is therefore critical for successful colonization and disease development. Our findings that Pc-Phr contributes to ROS tolerance and *in planta* survival suggest that photolyase-mediated processes directly facilitate the pathogen’s ability to overcome host-derived oxidative stress. Consistent with this observation, *phr*-deficient mutants in other bacterial species, such as *Neisseria gonorrhoeae*, exhibit increased sensitivity to oxidative stress (26).

In addition to ROS tolerance, Pc-Phr significantly contributed to resistance against UV-C and high-intensity BL, underscoring its pivotal role in environmental stress tolerance in PcJBC1. Similar roles for photolyases have been reported in *P. aeruginosa*, *P. syringae* (19), *P. syringae* pv. *syringae* B728a (21), *Shewanella oneidensis* (20), and *V. cholerae* (23). In some bacteria, photolyase function is integrated with broader stress response networks; for example, in *Acinetobacter* spp., UV resistance has been linked to elevated photolyase activity and coordinated induction of antioxidant defenses (69, 70); in *V. cholerae*, the DNA repair photolyase Cry1 is regulated by the RpoE–ChrR system, which also controls ROS defense genes such as glutaredoxin and glutathione S-transferase (23). The relatively low ROS levels observed in UV-treated wild-type and complemented strains in this study further suggest that efficient DNA repair by Pc-Phr stabilizes cellular metabolism and prevents excessive oxidative stress. This observation aligns with previous studies showing that bacterial responses to photo-oxidative stress involve coordinated activation of repair systems and antioxidant defenses to maintain redox balance (71, 72).

Finally, Pc-Phr was shown to contribute to virulence-associated traits, including host adhesion, colonization, and survival *in planta*. These traits are essential for disease development and have been linked to virulence in several plant-pathogenic bacteria, including *Agrobacterium tumefaciens* (73), *Pseudomonas syringae*, *Xanthomonas campestris*, and *Erwinia* spp. (74). The reduced virulence observed in the *phr*-deficient mutant indicates that efficient DNA repair and redox balance are critical determinants of bacterial fitness during host interaction. Collectively, our results indicate that Pc-Phr functions not only as a DNA repair enzyme but also as a key mediator linking environmental light cues to cellular stress responses. By coupling photoreactivation with oxidative stress control, Pc-Phr enhances bacterial survival under UV exposure and promotes pathogenicity in plant hosts. These results expand the current understanding of the functional scope of photolyases beyond genome maintenance and underscore their importance in environmental adaptation and host–pathogen interactions. Future investigations aimed at elucidating the regulatory networks governing *phr* expression and its integration with global stress response pathways will provide deeper insights into bacterial stress adaptation and pathogenicity.

### Conclusion

In this study, we identified and characterized Pc-Phr, a class I CPD photolyase from *P. cichorii* JBC1, and demonstrated its essential role in light-dependent DNA repair, stress tolerance, and pathogenicity. Pc-Phr exhibited hallmark photolyase properties, including blue light–driven photoreduction of flavin adenine dinucleotide (FAD) and efficient repair of UV-induced CPDs *in vitro* and *in vivo*. Loss of *phr* significantly compromised bacterial survival under UV-C, blue light, and oxidative stress conditions and markedly attenuated virulence in host plants, highlighting the importance of photorepair beyond genome maintenance. The light-inducible expression of *phr* further supports its role in coordinating adaptive responses to genotoxic stress. Collectively, these findings establish photolyase-mediated DNA repair as a key determinant of environmental fitness and virulence in a plant-pathogenic bacterium, underscoring the broader ecological importance of light-responsive stress adaptation mechanisms in plant–microbe interactions.

## SUPPLEMENTAL MATERIAL

Supplemental material aem.00255-26-s0001.pdf Fig. S1 to S6; Tables S1 to S4.

ASM does not own the copyrights to Supplemental Material that may be linked to, or accessed through, an article. The authors have granted ASM a non-exclusive, world-wide license to publish the Supplemental Material files. Please contact the corresponding author directly for reuse.
