# Supplementary material for: Role of photolyase in stress tolerance and virulence of plant-pathogenic bacterium Pseudomonas cichorii JBC1
Source: Appl Environ Microbiol. 2026 May 12;92(6):e00255-26. doi: 10.1128/aem.00255-26 (PMC13274461; doi:10.1128/aem.00255-26)
Supplement: Supplemental material — Fig. S1 to S6; Tables S1 to S4. [file aem.00255-26-s0001.pdf]

Supplementary Data

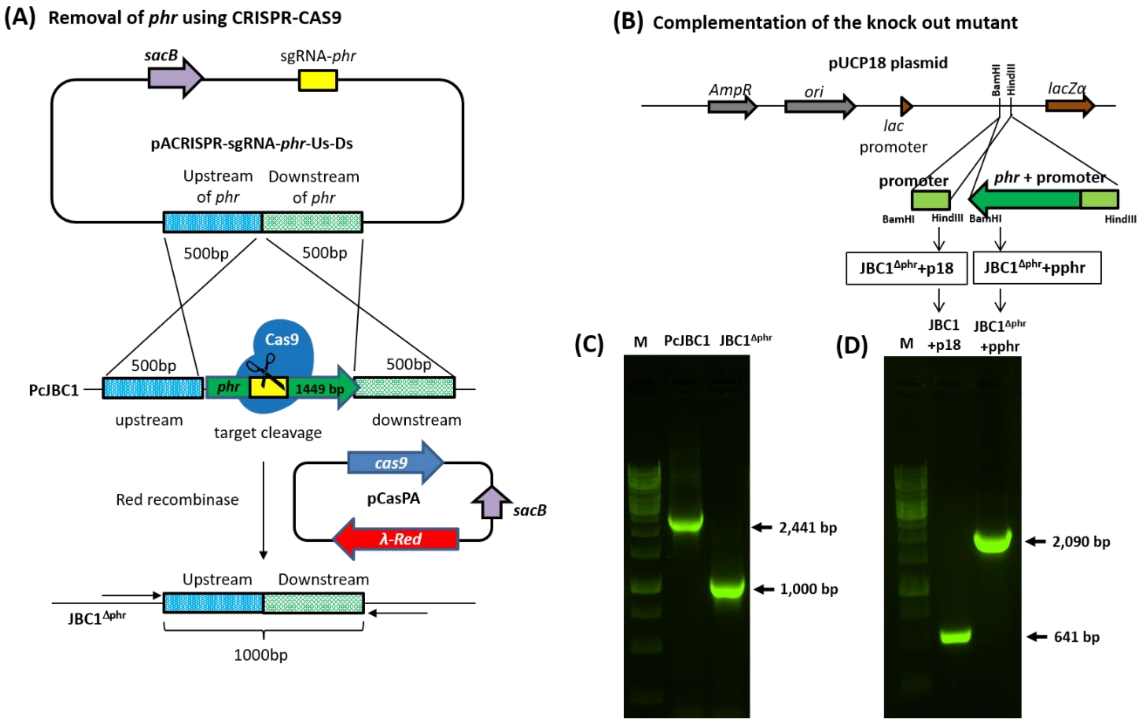

Fig. S1. Schematic workflow illustrating the construction of *phr*-deficient, vector control, and complementation strains in *Pseudomonas cichorii* JBC1. (A) Construction of *phr*-deficient strain (JBC1$^{\Delta phr}$) using the CRISPR/Cas9 system. (B) Generation of complemented strain carrying the *phr* gene and its native promoter (JBC1$^{\Delta phr}$ + pphr). (C, D) PCR-based verification of the JBC1$^{\Delta phr}$, vector control (*phr*-deficient strain harboring the empty pUCP18 vector with the native *phr* promoter; JBC1$^{\Delta phr}$+p18), and complementation strains. (C) PCR products amplified with PHR_Us_F and PHR_Ds_R primers. (D) PCR products amplified with M13F(-40) and M13pUC-R primers. Lane M, GeneRuler 1 kb DNA Ladder (Thermo Fisher Scientific Inc.).

```
PcJBC1        CAGGGCAAGTTCGGCGCGCAGATGGAGCGGGCGTTCTTTTATTCGTGGCTGCGCAGCAAG   60
JBC1Δphr      CAGGGCAAGTTCGGCGCGCAGATGGAGCGGGCGTTCTTTTATTCGTGGCTGCGCAGCAAG   60
              ************************************************************

PcJBC1        CTCGGCGCACGCATTTACCACAATAACCGGCAACTCAATGGCAAGCCGTTGCTACTGGTG   120
JBC1Δphr      CTCGGCGCACGCATTTACCACAATAACCGGCAACTCAATGGCAAGCCGTTGCTACTGGTG   120
              ************************************************************

PcJBC1        AACCAGTCCGACCTGCCGCTGGAGCCGCATTTGTGGCTCGTAGCCTGGCTGGTCAGCAGC   180
JBC1Δphr      AACCAGTCCGACCTGCCGCTGGAGCCGCATTTGTGGCTCGTAGCCTGGCTGGTCAGCAGC   180
              ************************************************************

PcJBC1        GCCGACTGCCCGGTGGAAGTCTTCGACTGGCCGCTGCCCGCAGGCGAACTGGCGCTGGCC   240
JBC1Δphr      GCCGACTGCCCGGTGGAAGTCTTCGACTGGCCGCTGCCCGCAGGCGAACTGGCGCTGGCC   240
              ************************************************************

PcJBC1        ACCGAATATCTGCAACCACGCGGCGTGCTGCTTTATTCCAGTAAATCGCTGAACCCAGCT   300
JBC1Δphr      ACCGAATATCTGCAACCACGCGGCGTGCTGCTTTATTCCAGTAAATCGCTGAACCCAGCT   300
              ************************************************************

PcJBC1        CAATTGCCCAGGTTATTGAGCAACATTTCCTGCCCGATCGTTCTCAGCGGATCAACGGTA   360
JBC1Δphr      CAATTGCCCAGGTTATTGAGCAACATTTCCTGCCCGATCGTTCTCAGCGGATCAACGGTA   360
              ************************************************************

PcJBC1        CAGATCCACTATGCCGATTTGCTCGTATGTGCGAACGAGGTCGCTGGATTGACCCTTGCT   420
JBC1Δphr      CAGATCCACTATGCCGATTTGCTCGTATGTGCGAACGAGGTCGCTGGATTGACCCTTGCT   420
              ************************************************************

PcJBC1        CAAGATCCGCTCAGTGCACAGATCGAATTGAACAGGCTCGGACTCATTTAAGGATTTCAC   480
JBC1Δphr      CAAGATCCGCTCAGTGCACAGATCGAATTGAACAGGCTCGGACTCATTTAAGGATTTCAC   480
              ************************************************************

PcJBC1        ATGCAACTGTTTTGGCTGCGTAGCGATTTGCGTGTTCACGACAATACTGCCCTGGCCGCT   540
JBC1Δphr      ------------------------------------------------------------   480

PcJBC1        GCCATGGAGCGGGGCCCGACGCTTGCGGTGTATCTGGTCAGTCCCACTCAATGGCAGAAT   600
JBC1Δphr      ------------------------------------------------------------   480

PcJBC1        CATGACGATGCCGCCTGCAAGGTGGATTTCTGGCTGCGCAATCTGGTCGAGCTGGAAAAA   660
JBC1Δphr      ------------------------------------------------------------   480

PcJBC1        GCCCTGGCCGGGCTGAATGTTCCCTTGCTGATCCGCAAGGCCGACACCTGGGACAAAGCC   720
JBC1Δphr      ------------------------------------------------------------   480

PcJBC1        CCTGAAGTGCTGGCAAAGCTCTGCGCGGAGCATGCCGTCGAAGGCGTGCACACCAATGAA   780
JBC1Δphr      ------------------------------------------------------------   480

PcJBC1        GAATACGGCATCAATGAAAGCAACCGCGATCAGGCGGTAGGCCAGGCGCTGGAAAAAGCC   840
JBC1Δphr      ------------------------------------------------------------   480

PcJBC1        GGTGTCCATTTCAACAGCTATCTGGATCAACTGCTGTTCAAGCCTGGCAGCATTCTGACC   900
JBC1Δphr      ------------------------------------------------------------   480

PcJBC1        AAGACCGGTGGCTACTTTCAGGTCTATAGCCAGTTCCGCAAAGTCTGCTATGCCCGCCTG   960
JBC1Δphr      ------------------------------------------------------------   480

PcJBC1        CACGAAGCGATGCCACGCCTCATCAATCTGCCAAACGCTCAGCAGCCGCTGTCGATCAAA   1020
JBC1Δphr      ------------------------------------------------------------   480

PcJBC1        AGCGATGCAGTGCCCGATCAGGTCGACGGTTTTGCCAGCCCATCCAAAGCACTGCGCGAA   1080
JBC1Δphr      ------------------------------------------------------------   480
```

```
PcJBC1          CTCTGGCCTGCTGGCGAACACGAAGCACGCCTGCGCCTGGCCACCTTCAGCGACGATCAG  1140
JBC1Δphr        ------------------------------------------------------------  480

PcJBC1          ATCCGCTATTACCAGAGCGAACGGGATTTCCCTGCAAAGCCCGGTACCAGCCAGTTATCG  1200
JBC1Δphr        ------------------------------------------------------------  480

PcJBC1          GCGTACCTCGCAGCCGGTGTGATTTCACCCCGCCAATGCCTGCACGCAGCACTGATGAGC  1260
JBC1Δphr        ------------------------------------------------------------  480

PcJBC1          AATGACGGAGAATTCGAGACCGGTAACACCGGGATAGTGACCTGGATCAACGAACTGTTG  1320
JBC1Δphr        ------------------------------------------------------------  480

PcJBC1          TGGCGGGAGTTCTACAAACACATTCTTGTGGGCTACCCTCGTGTTTCAAGGCATCGCGCA  1380
JBC1Δphr        ------------------------------------------------------------  477

PcJBC1          TTCCGCCCGGAAACCGAAGCCGTCAAATGGCGTAACGCGCCAACCGAACTGGCCGCGTGG  1440
JBC1Δphr        ------------------------------------------------------------  480

PcJBC1          CAGGAAGCCCGCACCGGTTTGCCGATCATCGATGCTGCCATGCGCCAGTTGCTGGAAACA  1500
JBC1Δphr        ------------------------------------------------------------  480

PcJBC1          GGCTGGATGCATAACCGACTAAGGATGGTCGTTGCAATGTTCCTGACCAAGAACCTGCTG  1560
JBC1Δphr        ------------------------------------------------------------  480

PcJBC1          ATTGACTGGCGCGAGGGCGAGCGCTTTTTCATGCGCCACCTGATCGACGGGGATCTGGCA  1620
JBC1Δphr        ------------------------------------------------------------  480

PcJBC1          GCCAATAACGGCGGCTGGCAGTGGAGTTCATCCACAGGCACCGACTCGGCCCCTTATTTC  1680
JBC1Δphr        ------------------------------------------------------------  480

PcJBC1          CGCATCTTCAATCCGCTGTCGCAATCGGAAAAATTCGATCCTGAAGGGCGCTTCATCAAG  1740
JBC1Δphr        ------------------------------------------------------------  480

PcJBC1          CACTGGTTGCCGGAACTGGCCAGCCTCAACAAGAAAGAGGTGCATAACCCGGCGCTTGTG  1800
JBC1Δphr        ------------------------------------------------------------  480

PcJBC1          GGCGGGCTGTTCGGTGTCGCCAACTATCCGTCCCCGATTGTAGACCTGAGTAAAAGCCGC  1860
JBC1Δphr        ------------------------------------------------------------  480

PcJBC1          GAGCGGGCCCTGGCCGCGTTCAAGGCCCTGCCCCATCGCCAGCCTGCTGCGGATGCGCTC  1920
JBC1Δphr        ------------------------------------------------------------  480

PcJBC1          CATGAGTGAATTCCTGCAACGCTTCGCCCATGACTTCGCAGCCCTGAACAAGGACAACCT  1980
JBC1Δphr        -ATGAGTGAATTCCTGCAACGCTTCGCCCATGACTTCGCAGCCCTGAACAAGGACAACCT  539
                 ***********************************************************

PcJBC1          TGAGCGAGTGGCCGACCTGTACAGCGACAACGTGTCGTTCACAGACCCGCTGCACCATAT  2040
JBC1Δphr        TGAGCGAGTGGCCGACCTGTACAGCGACAACGTGTCGTTCACAGACCCGCTGCACCATAT  599
                ************************************************************

PcJBC1          CCAGGGCCTGTCGGCCATGCAGGACTACTTCGCCCAGCTGTACAGCAATGTCAGTAACCT  2100
JBC1Δphr        CCAGGGCCTGTCGGCCATGCAGGACTACTTCGCCCAGCTGTACAGCAATGTCAGTAACCT  659
                ************************************************************

PcJBC1          ACATTTCGATTTCCACCACTTCGATGAAGTCAGGCCCGGCGAAGGCTATCTGGTCTGGAC  2160
JBC1Δphr        ACATTTCGATTTCCACCACTTCGATGAAGTCAGGCCCGGCGAAGGCTATCTGGTCTGGAC  719
                ************************************************************

PcJBC1          CATGAGTTATTCACACCCGCGCCTGAAAAAGGGTCGGGGCATCAAGGTCGAAGGCTGCTC  2220
JBC1Δphr        CATGAGTTATTCACACCCGCGCCTGAAAAAGGGTCGGGGCATCAAGGTCGAAGGCTGCTC  779
                ************************************************************

PcJBC1          GCATCTGCATTGGCACGAAAAGGTTTACCGGCATCGGGATTACTTCGATGCCGGCGCATT  2280
JBC1Δphr        GCATCTGCATTGGCACGAAAAGGTTTACCGGCATCGGGATTACTTCGATGCCGGCGCATT  839
                ************************************************************
```

```
PcJBC1        GCTCTATGAACACCTCCCCATCATGGGAGGCTTGATTGCCTGGCTGAAGAAGAGGTTGGC   2340
JBC1Δphr      GCTCTATGAACACCTCCCCATCATGGGAGGCTTGATTGCCTGGCTGAAGAAGAGGTTGGC   899
              ************************************************************

PcJBC1        ATGAACACGTCATATGCTCGTCGCATCTGGTTGACTGGCGCCAGCAGCGGTATTGGCCTC   2400
JBC1Δphr      ATGAACACGTCATATGCTCGTCGCATCTGGTTGACTGGCGCCAGCAGCGGTATTGGCCTC   959
              ************************************************************

PcJBC1        GCCCTGGCAAAAG       2413
JBC1Δphr      GCCCTGGCAAAAG       972
              *************
```

Fig. S2. Nucleotide sequence alignment of the *phr* gene region and its flanking sequences in *Pseudomonas cichorii* JBC1 and the *phr* deletion mutant (JBC1$^{\Delta phr}$). The alignment compares the genomic region containing the *phr* gene (highlighted in yellow) and its upstream and downstream flanking sequences (highlighted in gray) from the wild-type *P. cichorii* JBC1 (GenBank accession: CP007039.1) with the corresponding PCR-amplified region from the *phr*-deficient mutant (JBC1$^{\Delta phr}$), obtained using primers PHR_Us_F and PHR_Ds_R. Sequence alignment was performed using Clustal Omega. Asterisks (*) indicate conserved nucleotides, while mismatches or gaps indicate deleted regions. The absence of the *phr* coding sequence in JBC1$^{\Delta phr}$ confirms successful gene deletion.

.

```
PcJBC1                  ----------------CCGAGCTGGAAAAGCGCTGGCAGGGCAAGTTCGGCGCGCAGATG  44
JBC1Δphr+pphr           GGCCAGTGCCAAGCTTCCGAGCTGGAAAAGCGCTGGCAGGGCAAGTTCGGCGCGCAGATG  60
                                        ********************************************

PcJBC1                  GAGCGGGCGTTCTTTTATTCGTGGCTGCGCAGCAAGCTCGGCGCACGCATTTACCACAAT  104
JBC1Δphr+pphr           GAGCGGGCGTTCTTTTATTCGTGGCTGCGCAGCAAGCTCGGCGCACGCATTTACCACAAT  120
                        ************************************************************

PcJBC1                  AACCGGCAACTCAATGGCAAGCCGTTGCTACTGGTGAACCAGTCCGACCTGCCGCTGGAG  164
JBC1Δphr+pphr           AACCGGCAACTCAATGGCAAGCCGTTGCTACTGGTGAACCAGTCCGACCTGCCGCTGGAG  180
                        ************************************************************

PcJBC1                  CCGCATTTGTGGCTCGTAGCCTGGCTGGTCAGCAGCGCCGACTGCCCGGTGGAAGTCTTC  224
JBC1Δphr+pphr           CCGCATTTGTGGCTCGTAGCCTGGCTGGTCAGCAGCGCCGACTGCCCGGTGGAAGTCTTC  240
                        ************************************************************

PcJBC1                  GACTGGCCGCTGCCCGCAGGCGAACTGGCGCTGGCCACCGAATATCTGCAACCACGCGGC  284
JBC1Δphr+pphr           GACTGGCCGCTGCCCGCAGGCGAACTGGCGCTGGCCACCGAATATCTGCAACCACGCGGC  300
                        ************************************************************

PcJBC1                  GTGCTGCTTTATTCCAGTAAATCGCTGAACCCAGCTCAATTGCCCAGGTTATTGAGCAAC  344
JBC1Δphr+pphr           GTGCTGCTTTATTCCAGTAAATCGCTGAACCCAGCTCAATTGCCCAGGTTATTGAGCAAC  360
                        ************************************************************

PcJBC1                  ATTTCCTGCCCGATCGTTCTCAGCGGATCAACGGTACAGATCCACTATGCCGATTTGCTC  404
JBC1Δphr+pphr           ATTTCCTGCCCGATCGTTCTCAGCGGATCAACGGTACAGATCCACTATGCCGATTTGCTC  420
                        ************************************************************

PcJBC1                  GTATGTGCGAACGAGGTCGCTGGATTGACCCTTGCTCAAGATCCGCTCAGTGCACAGATC  464
JBC1Δphr+pphr           GTATGTGCGAACGAGGTCGCTGGATTGACCCTTGCTCAAGATCCGCTCAGTGCACAGATC  480
                        ************************************************************

PcJBC1                  GAATTGAACAGGCTCGGACTCATTTAAGGATTTCACATGCAACTGTTTTGGCTGCGTAGC  524
JBC1Δphr+pphr           GAATTGAACAGGCTCGGACTCATTTAAGGATTTCACATGCAACTGTTTTGGCTGCGTAGC  540
                        ************************************************************

PcJBC1                  GATTTGCGTGTTCACGACAATACTGCCCTGGCCGCTGCCATGGAGCGGGGCCCGACGCTT  584
JBC1Δphr+pphr           GATTTGCGTGTTCACGACAATACTGCCCTGGCCGCTGCCATGGAGCGGGGCCCGACGCTT  600
                        ************************************************************

PcJBC1                  GCGGTGTATCTGGTCAGTCCCACTCAATGGCAGAATCATGACGATGCCGCCTGCAAGGTG  644
JBC1Δphr+pphr           GCGGTGTATCTGGTCAGTCCCACTCAATGGCAGAATCATGACGATGCCGCCTGCAAGGTG  660
                        ************************************************************

PcJBC1                  GATTTCTGGCTGCGCAATCTGGTCGAGCTGGAAAAAGCCCTGGCCGGGCTGAATGTTCCC  704
JBC1Δphr+pphr           GATTTCTGGCTGCGCAATCTGGTCGAGCTGGAAAAAGCCCTGGCCGGGCTGAATGTTCCC  720
                        ************************************************************

PcJBC1                  TTGCTGATCCGCAAGGCCGACACCTGGGACAAAGCCCCTGAAGTGCTGGCAAAGCTCTGC  764
JBC1Δphr+pphr           TTGCTGATCCGCAAGGCCGACACCTGGGACAAAGCCCCTGAAGTGCTGGCAAAGCTCTGC  780
                        ************************************************************

PcJBC1                  GCGGAGCATGCCGTCGAAGGCGTGCACACCAATGAAGAATACGGCATCAATGAAAGCAAC  824
JBC1Δphr+pphr           GCGGAGCATGCCGTCGAAGGCGTGCACACCAATGAAGAATACGGCATCAATGAAAGCAAC  840
                        ************************************************************

PcJBC1                  CGCGATCAGGCGGTAGGCCAGGCGCTGGAAAAAGCCGGTGTCCATTTCAACAGCTATCTG  884
JBC1Δphr+pphr           CGCGATCAGGCGGTAGGCCAGGCGCTGGAAAAAGCCGGTGTCCATTTCAACAGCTATCTG  900
                        ************************************************************

PcJBC1                  GATCAACTGCTGTTCAAGCCTGGCAGCATTCTGACCAAGACCGGTGGCTACTTTCAGGTC  944
JBC1Δphr+pphr           GATCAACTGCTGTTCAAGCCTGGCAGCATTCTGACCAAGACCGGTGGCTACTTTCAGGTC  960
                        ************************************************************

PcJBC1                  TATAGCCAGTTCCGCAAAGTCTGCTATGCCCGCCTGCACGAAGCGATGCCACGCCTCATC  1004
JBC1Δphr+pphr           TATAGCCAGTTCCGCAAAGTCTGCTATGCCCGCCTGCACGAAGCGATGCCACGCCTCATC  1020
                        ************************************************************

PcJBC1                  AATCTGCCAAACGCTCAGCAGCCGCTGTCGATCAAAAGCGATGCAGTGCCCGATCAGGTC  1064
JBC1Δphr+pphr           AATCTGCCAAACGCTCAGCAGCCGCTGTCGATCAAAAGCGATGCAGTGCCCGATCAGGTC  1080
                        ************************************************************

PcJBC1                  GACGGTTTTGCCAGCCCATCCAAAGCACTGCGCGAACTCTGGCCTGCTGGCGAACACGAA  1124
JBC1Δphr+pphr           GACGGTTTTGCCAGCCCATCCAAAGCACTGCGCGAACTCTGGCCTGCTGGCGAACACGAA  1140
                        ************************************************************

PcJBC1                  GCACGCCTGCGCCTGGCCACCTTCAGCGACGATCAGATCCGCTATTACCAGAGCGAACGG  1184
JBC1Δphr+pphr           GCACGCCTGCGCCTGGCCACCTTCAGCGACGATCAGATCCGCTATTACCAGAGCGAACGG  1200
                        ************************************************************

PcJBC1                  GATTTCCCTGCAAAGCCCGGTACCAGCCAGTTATCGGCGTACCTCGCAGCCGGTGTGATT  1244
JBC1Δphr+pphr           GATTTCCCTGCAAAGCCCGGTACCAGCCAGTTATCGGCGTACCTCGCAGCCGGTGTGATT  1260
                        ************************************************************
```

```
PcJBC1              TCACCCCGCCAATGCCTGCACGCAGCACTGATGAGCAATGACGGAGAATTCGAGACCGGT  1304
JBC1Δphr+pphr       TCACCCCGCCAATGCCTGCACGCAGCACTGATGAGCAATGACGGAGAATTCGAGACCGGT  1320
                    ************************************************************

PcJBC1              AACACCGGGATAGTGACCTGGATCAACGAACTGTTGTGGCGGGAGTTCTACAAACACATT  1364
JBC1Δphr+pphr       AACACCGGGATAGTGACCTGGATCAACGAACTGTTGTGGCGGGAGTTCTACAAACACATT  1380
                    ************************************************************

PcJBC1              CTTGTGGGCTACCCTCGTGTTTCAAGGCATCGCGCATTCCGCCCGGAAACCGAAGCCGTC  1424
JBC1Δphr+pphr       CTTGTGGGCTACCCTCGTGTTTCAAGGCATCGCGCATTCCGCCCGGAAACCGAAGCCGTC  1440
                    ************************************************************

PcJBC1              AAATGGCGTAACGCGCCAACCGAACTGGCCGCGTGGCAGGAAGCCCGCACCGGTTTGCCG  1484
JBC1Δphr+pphr       AAATGGCGTAACGCGCCAACCGAACTGGCCGCGTGGCAGGAAGCCCGCACCGGTTTGCCG  1500
                    ************************************************************

PcJBC1              ATCATCGATGCTGCCATGCGCCAGTTGCTGGAAACAGGCTGGATGCATAACCGACTAAGG  1544
JBC1Δphr+pphr       ATCATCGATGCTGCCATGCGCCAGTTGCTGGAAACAGGCTGGATGCATAACCGACTAAGG  1560
                    ************************************************************

PcJBC1              ATGGTCGTTGCAATGTTCCTGACCAAGAACCTGCTGATTGACTGGCGCGAGGGCGAGCGC  1604
JBC1Δphr+pphr       ATGGTCGTTGCAATGTTCCTGACCAAGAACCTGCTGATTGACTGGCGCGAGGGCGAGCGC  1620
                    ************************************************************

PcJBC1              TTTTTCATGCGCCACCTGATCGACGGGGATCTGGCAGCCAATAACGGCGGCTGGCAGTGG  1664
JBC1Δphr+pphr       TTTTTCATGCGCCACCTGATCGACGGGGATCTGGCAGCCAATAACGGCGGCTGGCAGTGG  1680
                    ************************************************************

PcJBC1              AGTTCATCCACAGGCACCGACTCGGCCCCTTATTTCCGCATCTTCAATCCGCTGTCGCAA  1724
JBC1Δphr+pphr       AGTTCATCCACAGGCACCGACTCGGCCCCTTATTTCCGCATCTTCAATCCGCTGTCGCAA  1740
                    ************************************************************

PcJBC1              TCGGAAAAAATTCGATCCTGAAGGGCGCTTCATCAAGCACTGGTTGCCGGAACTGGCCAGC  1784
JBC1Δphr+pphr       TCGGAAAAAATTCGATCCTGAAGGGCGCTTCATCAAGCACTGGTTGCCGGAACTGGCCAGC  1800
                    ************************************************************

PcJBC1              CTCAACAAGAAAGAGGTGCATAACCCGGCGCTTGTGGGCGGGCTGTTCGGTGTCGCCAAC  1844
JBC1Δphr+pphr       CTCAACAAGAAAGAGGTGCATAACCCGGCGCTTGTGGGCGGGCTGTTCGGTGTCGCCAAC  1860
                    ************************************************************

PcJBC1              TATCCGTCCCCGATTGTAGACCTGAGTAAAAGCCGCGAGCGGGCCCTGGCCGCGTTCAAG  1904
JBC1Δphr+pphr       TATCCGTCCCCGATTGTAGACCTGAGTAAAAGCCGCGAGCGGGCCCTGGCCGCGTTCAAG  1920
                    ************************************************************

PcJBC1              GCCCTGCCCCATCGCCAGCCTGCTGCGGATGCGCTCCATGAGTGA---------------  1949
JBC1Δphr+pphr       GCCCTGCCCCATCGCCAGCCTGCTGCGGATGCGCTCCATGAGTGAGGATCCCCGGGTACC  1980
                    *********************************************

PcJBC1              ---------------------     1949
JBC1Δphr+pphr       GAGCTCGAATTCGTAATCATG     2001
```

Fig. S3. Nucleotide sequence alignment of the *phr* gene region (highlighted in yellow) and its upstream sequences (gray) from *Pseudomonas cichorii* JBC1 (GenBank: CP007039.1) with the corresponding PCR-amplified sequence from the complemented strain (JBC1$^{\Delta phr}$+pphr), generated using M13F(-40) and M13pUC-R primers. Clustal Omega was used for alignment. Identical nucleotides are indicated by asterisks (*), and mismatches are shown as blank spaces. The results confirm successful complementation of the *phr* gene in the complemented strain.

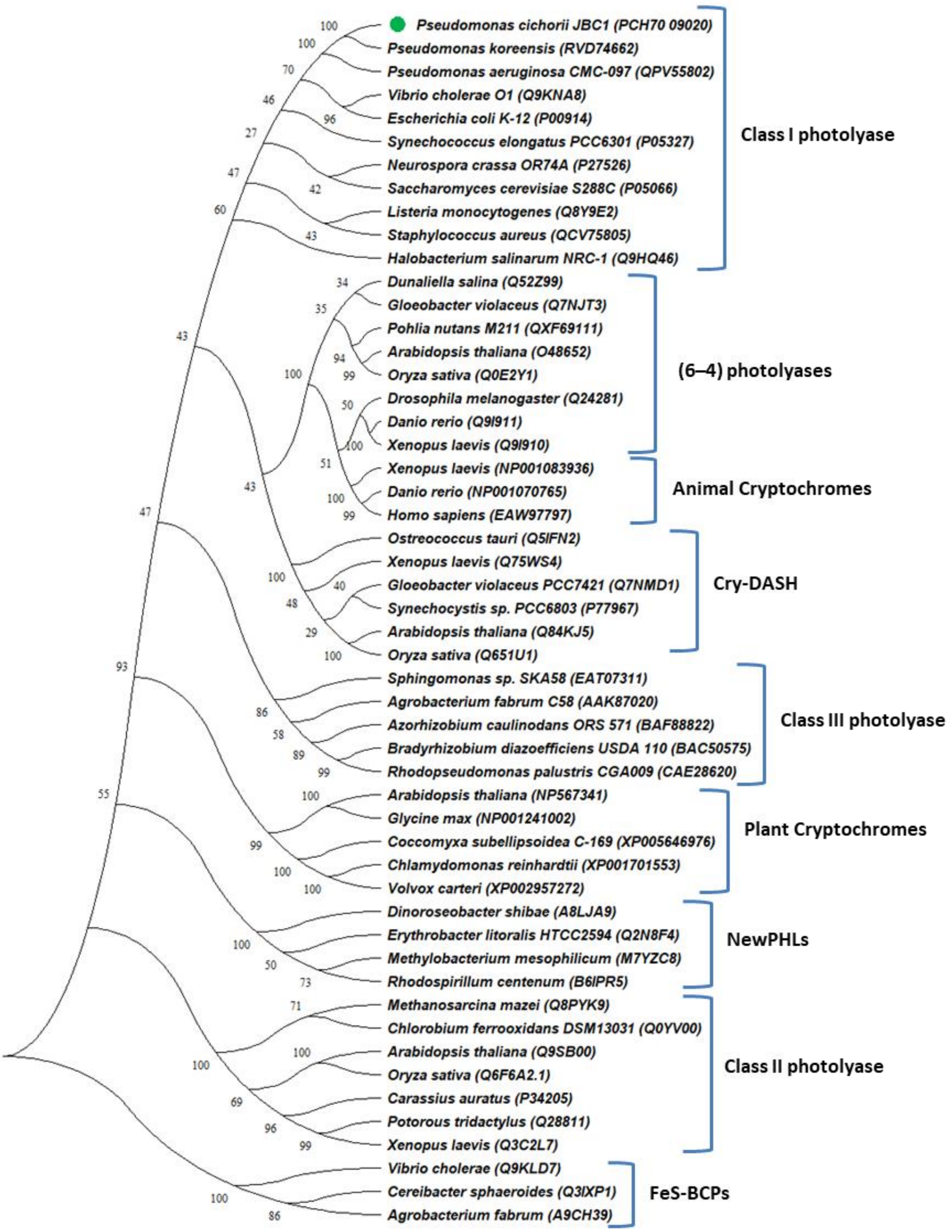

Fig. S4. Comparison of the evolutionary relationships and protein conservation of Pc-Phr from *Pseudomonas cichorii* JBC1 and other photolyases and cryptochromes. An unrooted phylogenetic tree of cryptochrome/photolyase family (CPF) proteins was constructed using the neighbor-joining method. The bootstrap probabilities based on 1,000 replicates are shown as percentage frequencies at the corresponding nodes. Amino acid sequences from various species are retrieved from the NCBI protein database with accession numbers provided in parentheses.

```
Pc-Phr          ----------------------------MQLFWLRSDLRVHDNTALAAAM-------ERG 25
Pk-CPDI         ----------------------------MQLIWLRSDLRQHDNTALAAAA-------ARG 25
Vc-CPDI         ----------------------------MRLVWFRRDLRSFDNTALTAAL------NSGD 26
Ec-CPDI         --------------------------MTTHLVWFRQDLRLHDNLALAAAC----R-NSSA 29
Af-CPDIII       ----------------------MSLKTAPVIVWFRKDLRLSDNLALLAAV------EHGG 32
Ac-CPDIII       -----------------------MSAAGTAVIWFRDDLRLADNPALAAAA------ASGR 31
Bd-CPDIII       ------------------------------------------------------------ 0
Ot-DASH         ------------------------MGRTRVVIWFRNDLRLLDNACVARAATLASE-SSDV 35
Gv-DASH         ------------------------MSTKTVLVWYRNDLRVHDHEPLTSAL------HKNA 30
Ss-DASH         ----------------------MKHVPPTVLVWFRNDLRLHDHEPLHRAL------KSGL 32
Dm-[6-4]Phr     ----------------------MDSQRSTLVHWFRKGLRLHDNPALSHIFTAANAAPGKY 38
Os-[6-4]Phr     ---------------MDAAATAATATAAAAMVWFRKGLRVHDNPALDAARRGAA----A 41
Gv-[6-4]Phr     --------------------------MIRSLVWFRKGLRLHDNPALLDAAR--DA----A 28
At-CPDII        MASTVSVQPGRIRILKKGSWQPLDQTVGPVVYWMFRDQRLKDNWALIHAVDLANR--TNA 58
Cf-CPDII        -----MIDPRRITLLNNC-----RDASGPVIYWMSRDQRVRHNWALLFARKKAEQ--LQQ 48
Mm-CPDII        ----MIMNPKRIRALKSG-----KQGDGPVVYWMSRDQRAEDNWALLFSRAIAKE--ANV 49

Pc-Phr          PTLAVYLVSPTQWQNH----DD-----AACKVDFWLRNLVELEKALAGLNVPLLIRKADT 76
Pk-CPDI         PTVAVYLLSPRQWLEH----DD-----AACKVDFWLRNLRELSSALGELNIPLLIRTADH 76
Vc-CPDI         PVAAMYIATPEQWHQH----HL-----APIQADLIWRRLAELQQELAALNVPLFYQQVAD 77
Ec-CPDI         RVLALYIATPRQWATH----NM-----SPRQAELINAQLNGLQIALAEKGIPLLFREVDD 80
Af-CPDIII       PVIPVYIRE---KSAG----PL-----GGAQEWWLHHSLAALSSSLEKAGGRLVLASGD- 79
Ac-CPDIII       RVLALYVLDEDSPGVR----AL-----GGAALWWLAGSLRALKAELGQHGVPLVLRRGE- 81
Bd-CPDIII       --------MLDETAGR----AP-----GGAARWWLAQSLRALGAEIAARGGSLILRKGP- 42
Ot-DASH         EVVPVYVFDETYFKPS----KRGLARFGAGRGKFTLECVGDLKTSLRALGSDLLVRCGK- 90
Gv-DASH         RVVALYCFDPRQFGKA----PFGFEKTGPFRARFLLESVADLRRSLRQLGSDLLVRRGH- 85
Ss-DASH         AITAVYCYDPRQFAQT----HQGFAKTGPWRSNFLQQSVQNLAESLQKVGNKLLVTTGL- 87
Dm-[6-4]Phr     FVRPIFILDPGILDWM---------QVGANRWRFLQQTLEDLDNQLRKLNSRLFVVRGKP 89
Os-[6-4]Phr     RLYPVFVLDPRYLRPDQAAPSPGSARAGVARVRFLLESLSDLDARLRRLGSRLLLLRARD 101
Gv-[6-4]Phr     RLYPLFIVDPWFVNPE---------RVGVNRMRFLLESLGEIDGNLRRLGSRLIVLQGRP 79
At-CPDII        PVAVVFNLFDQFLDA------------KARQLGFMLKGLRQLHHQIDSLQIPFFLLQGD- 105
Cf-CPDII        PLKVVFTLAPSFLNA------------PLRHYDFMLKGLHEVERALRRLNIPFYLVQGE- 95
Mm-CPDII        PVVVVFCLTDEFLEA------------GIRQYEFMLKGLQELEVSLSRKKIPSFFLRGD- 96
                                                      :  :   :

Pc-Phr          WDKAPEVLAKLCAEH--AVEGVHTNEEYGINESNRDQAVGQ------ALEKAGVHFNSYL 128
Pk-CPDI         WDQAPVVLVELCRQL--NVEAVHVNEEYGVHESRRDAAVAQ------ALKTTGIAFHSYL 128
Vc-CPDI         FQAAAVAVSQLAKTL--NATQVLANRDYELDEQQRDQLAQQ------LLSEQGIIWSAFD 129
Ec-CPDI         FVASVEIVKQVCAEN--SVTHLFYNYQYEVNERARDVEVER------A--LRNVVCEGFD 130
Af-CPDIII       ---AERILRDLISET--GADTVVWNRRYDPTGMATDKALKQ------KLRDDGLTVRSFS 128
Ac-CPDIII       ---AGRIVPDVARAA--GADLVAFNWRAGKAEQAVDEAVAR------HLSAAGVTVERTL 130
Bd-CPDIII       ---AAGVIPEEARES--GARAVYWNGIAQAPHQAIERRLEA------ALAKLGVDSQSFP 91
Ot-DASH         ---SRDVIAELTLTGANDRTIILTQTEVTSEETEMDVAVERATRERARGGAASATMERHW 147
Gv-DASH         ---PEEVIPALVSEL--EIAAVHYHGEVTSEELVVERDLQA------ALAPLNVPVRSFW 134
Ss-DASH         ---PEQVIPQIAKQI--NAKTIYYHREVTQEELDVERNLVK------QLTILGIEAKGYW 136
Dm-[6-4]Phr     AE----VFPRIFKSW--RVEMLTFETDIEPYSVTRDAAVQK------LAKAEGVRVETHC 137
Os-[6-4]Phr     DGDVAGTVCAALKDW--NIGKLCFESDTEPYALARDKKVMD------FAAASGIDVFSPV 153
Gv-[6-4]Phr     QE----VLERVLSRW--QIGRLCFERDTEPYARRRDEAIRS------MAERVGVRVISPT 127
At-CPDII        ---AKETIPNFLTEC--GASHLVTDFSPLREIRRCKDEVVK-------RTSDSLAIHEVD 153
Cf-CPDII        ---PEIELPRFAREM--KAGAVVTDFSPLKISREWKRRVGA-------H--LPLPLYEVD 141
Mm-CPDII        ---PGEKISRFVKDY--NAGTLVTDFSPLRIKNQWIEKVIS-------G--ISIPFFEVD 142
                       .             :  .

Pc-Phr          DQLLFKPGSI----LTKTGGYFQVYSQFRKVCYARL-HEA----MPRLINLPNAQQPLSI 179
Pk-CPDI         DQLLFKPGTV----LTKTETYFQVFSQFRKVCYERL-HRS----VPGLIKAPGKQAKLNI 179
Vc-CPDI         DKCVLPPGSV----RTKQGEFFKVFTPFKRAWLTLF-QPP----VIGKNRPVALWNVPSA 180
Ec-CPDI         DSVILPPGAV----MTGNHEMYKVFTPFKNAWLKRL-REG----MPECVAAPKVR----S 177
Af-CPDIII       GQLLHEPSRL----QTKSGGPYRVYTPFWRALEGSD-EPHAPADPPKSLTAPKVWPKSEK 183
Ac-CPDIII       GHLLHAPGSV----RGTGGGLPRTFSSFMRAAVKGR-DIGRTLPVPRLNG--AAPLPCDD 183
Bd-CPDIII       GDLLVPPSAI----RNKEGRGLRVFTPFWRRVLSLG-DPPKPLPAPKQLR-PGPKIVSDR 145
Ot-DASH         GSTLYHIDDVPFDVTSGLSDLPDVFTPFRNKVESKC-KVRDVIPAPTANELGHVPASVEG 206
Gv-DASH         GTTLVHPDDLPFAI----EAIPELFTDFRKQVERSA-AINPPLPAPAK--LPPLP--AVD 185
Ss-DASH         GSTLCHPEDLPFSI----QDLPDLFTKFRKDIEKKKISIRPCFFAPSQ--LLPSP--NIK 188
Dm-[6-4]Phr     SHTIYNPELV---KAKNLGKAPITYQKFLGIVEQLK-VPK-------------VLGVPEK 180
Os-[6-4]Phr     SHTLFDPAEI---IEKNGGRPPMTYQSFVAIAG----EPP-------------EPIME-E 192
Gv-[6-4]Phr     AHTLYDPDEL---IELGRGKVPTTYGAFGRLAAKLG-EPD-------------APVA--S 168
At-CPDII        AHNVVPMWAA----SSKLEYSARTIRGKINKLLPDYLIEFPKLEPPKKK---WTGMMDKK 206
Cf-CPDII        AHNIVPCRIA----SAKQEYAARTFRPKIKSLLGELLTGFPQLEPLEAT---TES----H 190
Mm-CPDII        AHNVVPCWEA----SQKHEYAAHTFRPKLYALLPEFLEEFPELEPNSVT---PEL----S 191
                   :
```

```
Pc-Phr           KSDAVPDQV-DGFAS-PSKAL------------------RELWPAGEHEARLR----LAT 215
Pk-CPDI          DSDPVPASV-EGFAA-PSESL------------------RTLWPAGEQQARRR----LDT 215
Vc-CPDI          LAELVWHPE-QAFDY-PRI--------------------DSTPWAADFETVRAQ----LRD 215
Ec-CPDI          SGSIEPSPS-ITLNY-PRQSF------------------DTAHFPVEEKAAIAQ----LRQ 214
Af-CPDIII        LSNWKLLPT-KP--D-WAKD------------------FSDIWTPGETGALDK----LDD 217
Ac-CPDIII        LEEWALEPT-QP--D-WAGG------------------LRTTWHPGEAAARAR----LEA 217
Bd-CPDIII        LESWQLAPT-KP--D-WAGG------------------LRERWTPGEASARAR----LRD 179
Ot-DASH          FEWMPKPSD-LPFAS-SEIAMDCDK----RIKDCLDERSVLDFKGGESNALAR----VKY 256
Gv-DASH          PGEIPQLAD-LGLE-----------------SPVTDERAVLQFKGGETSGLAR----LEE 223
Ss-DASH          LELTAPPP--EFFP-----------------QINFDHRSVLAFQGGETAGLAR----LQD 225
Dm-[6-4]Phr      LKKMPTPPK-DEVEQKDSAAYDCPTIKQLVKRP--EELGPNKFPGGETEALRR----MEE 233
Os-[6-4]Phr      YSEL--PPV-GDTG--EYELLPVPRVEELGYGDISQEDLS-LFRGGETEALKR----MRE 242
Gv-[6-4]Phr      PSHL--PPP-GELD--A--DYGIPTLAELGYPDPECPSRGIIPPGGEGEGLRR----LHV 217
At-CPDII         LVDWD--SL-------------I--DKVVREG--AEVPEIEWCVPGEDAGIEVLMGNKDG 247
Cf-CPDII         PVDWS--SI----------------SCSLKAD--HRVAPVEWLKPGEEAAANC----LDT 226
Mm-CPDII         AGAGMVETLSDVLETGVKAL--LPERALLKNK--DPLFEPWHFEPGEKAAKKV----MES 243

Pc-Phr           FS--DDQIRYYQSERDF---PAKPGTSQLSAYLAAGVISPRQCLHAALMSNDGEFETGNT 270
Pk-CPDI          FA--DAQIDYYQSERDF---PAKPGTSQLSAYLAAGVISPRQCLHAALQSNQGEFDSGKV 270
Vc-CPDI          FC--RERVQDYHQARDF---PAREGTSSLSPYLAIGVLSARQCVARLYHESSMGEL--SE 268
Ec-CPDI          FC--QNGAGEYEQQRDF---PAVEGTSRLSASLATGGLSPRQCLHRLLAEQPQALD--GG 267
Af-CPDIII        FI--DGALKGYEEGRDF---PAKPATSLLSPHLAAGEISPAAVWHATKGL--SRHI-ASN 269
Ac-CPDIII        FL--ADGLAGYGEGRDR---PDQTHVSRLSPYLRFGEISPRQVLLAALAAGDAGTV-PAK 271
Bd-CPDIII        FL--KTIARGYAGDRDR---PDRVGTSGLSPHLRFGELSPRQVWHAARFAAAEDAA-LGP 233
Ot-DASH          YLWESDRLATYFETRNG--MLGGDYSTKLAPWLALGCVSPRHVVSEIRRYESERVE--NK 312
Gv-DASH          YFWQKSLLKSYKQTRNG--MLGADYSSKFSAWLALGCLSARYIHEQVQTYETKRIK--ND 279
Ss-DASH          YFWHGDRLKDYKETRNG--MVGADYSSKFSPWLALGCLSPRFIYQEVKRYEQERVS--ND 281
Dm-[6-4]Phr      SLKDEIWVARFEKPNTAPNSL-EPSTTVLSPYLKFGCLSARLFNQKLKEIIKRQPK-HSQ 291
Os-[6-4]Phr      SLHDKEWVAKFEKPKGDPSAFLKPATTVLSPYLKFGCLSSRYFYHCIQDIYRSTKK-HTN 301
Gv-[6-4]Phr      YLSDRQRSAGFAKPDTDPTAFDPPSTTALGAHLKFGCLSARTFYAEVQKVYREVGE-HTE 276
At-CPDII         FL--TKRLKNYSTDRNNPIKP--KALSGLSPYLHFGQVSAQRCALEARKVRSTSPQ---- 299
Cf-CPDII         FL--EKRLSSYAELRNDPNS---GVLSNLSPYLHFGQISAQYIALRVSESRM-PDE---- 276
Mm-CPDII         FI--ADRLDSYGALRNDPTK---NMLSNLSPYLHFGQISSQRVVLEVEKAES-NPG---- 293
                           :               ::.   *  * :*

Pc-Phr           GIVTWI-NELLWREFYKHILVGYPRVSRHRAFRPETEAV------KWRNAPTELAAWQEA 323
Pk-CPDI          GAVTWI-NELLWREFYKHILVGYPRVSRHRAFRPETEAV------AWRNAPDELAAWQEA 323
Vc-CPDI          GAQVWL-SELIWREFYQHLVAIEPNLSKSRDFVEWGARL------EWWNDNEKFQLWCEG 321
Ec-CPDI          AGSVWL-NELIWREFYRHLITYHPSLCKHRPFIAWTDRV------QWQSNPAHLQAWQEG 320
Af-CPDIII        DISRFR-KEIVWREFCYHLLFHFPELGEK-NWNDSFDAF------SWRDDEKSFKAWTRG 321
Ac-CPDIII        DVQKFE-AELYWREFSHHLLFAVPDLARR-NLQASFDAF------PWRECPGDLKKWQKG 323
Bd-CPDIII        GIEKFL-SELGWREFCRHLLHDHPDLATE-NLQTNFDGF------PWQSDGKVLAAWQRG 285
Ot-DASH          STYWVI-FELIWRDFFKFFALKHGNKIFHLD-GTAGRRA------SWKRDEKILKAWKTG 364
Gv-DASH          STYWLI-FELLWRDYFRFIAAKHGDRLFYTA-GLRGLDI------PWKEDWERFELWRTG 331
Ss-DASH          STHWLI-FELLWRDFFRFVAQKYGNKLFNRG-GLLNKNF------PWQEDQVRFELWRSG 333
Dm-[6-4]Phr      PPVSLI-GQLMWREFYYTVAAAEPNFDRMLG-NVYCMQI------PWQEHPDHLEAWTHG 343
Os-[6-4]Phr      PPVSLT-GQLLWRDFFYTVAFGTPNFDQMKG-NKICKQI------PWTENEELFPAWRDG 353
Gv-[6-4]Phr      PPMSLI-GQILWREFFYTVGYATPNYDRIEG-NPVCRQI------PWDDNPEYLAAWSEA 328
At-CPDII         AVDTFLEELIVRRELSDNFCYYQPHYDSLKGAWEWARKSLMDHASDKREHIYSLEQLEKG 359
Cf-CPDII         SRSAFLEELIVRRELSDNYCFYNDRYDSFDGSPTWAKESLMNHRNDHREYLYTADEFATA 336
Mm-CPDII         SKKAFLDEILIWKEISDNFCYYNPGYDGFESFPSWAKESLNAHRNDVRSHIYTLEEFEAG 353
                          :  ::                                             .

Pc-Phr           RTGLPIIDAAMRQLLETGWMHNRLRMVVAMFLTK-NLLIDWREGERFFM--------RHL 374
Pk-CPDI          RTGLPIIDAAMRQLLETGWMHNRLRMVVAMFLTK-NLLIDWREGERFFM--------RHL 374
Vc-CPDI          KTGYPIVDAAMRQLNQTGWMHNRLRMIVASFLTK-DLHIDWRWGERYFM--------SRL 372
Ec-CPDI          KTGYPIVDAAMRQLNSTGWMHNRLRMITASFLVK-DLLIDWREGERYFM--------SQL 371
Af-CPDIII        MTGYPIVDAGMRQLWQHGTMHNRVRMIVASFLIK-HLLIDWRKGEKWFR--------DTL 372
Ac-CPDIII        RTGYPIVDAGMRQLWQTGWMHNRVRMIVASFLCK-HLLLPWQDGELWFW--------DTL 374
Bd-CPDIII        RTGYPIVDAGLRELWHTGVMHNRVRMVVASFLVK-HLLIDWRDGEAWFW--------DTL 336
Ot-DASH          TTGYPLIDANMRELAATGFMSNRGRQNVASWLAL-DAGIDWRHGADWFE--------HHL 415
Gv-DASH          QTGFPLVDANMRELAATGFMSNRGRQNVASFLTK-NLGIHWHMGAEWFE--------SRL 382
Ss-DASH          QTGYPLVDANMRELNLTGFMSNRGRQNVASFLCK-NLGIDWRWGAEWFE--------SCL 384
Dm-[6-4]Phr      RTGYPFIDAIMRQLRQEGWIHHLARHAVACFLTRGDLWISWEEGQRVFE--------QLL 395
Os-[6-4]Phr      RTGYPWIDAIMIQLRKWGWMHHLARHSVACFLTRGDLFIHWEKGRDVFE--------RLL 405
Gv-[6-4]Phr      RTGFPWIDAAMTQLRTEGWLHHLSRHAVACFLTRGDLWVSWEKGQAVFE--------RLL 380
At-CPDII         LTADPLWNASQLEMVYQGKMHGFMRMYWAKK------ILEWTKGPEEALSISIYLNNKYE 413
Cf-CPDII         KTHDRLWNAAQLELVTTGKIHGYMRMYWAKK------ILEWSESAEQAFETAMALNDRYA 390
Mm-CPDII         KTHDPLWNASQMELLSTGKMHGYMRMYWAKK------ILEWSESPEKALEIAICLNDRYE 407
                  *     :*   ::   * :    *   *         : *  .
```

```
Pc-Phr           IDGDLAANNGGWQWSSSTGTDSA-------PYFRIFNPLSQSEKFDPEGRFIKHWLPELA 427
Pk-CPDI          IDGDLAANNGGWQWSASTGTDSA-------PYFRIFNPLSQSEKFDSEGLFIKHWLPELA 427
Vc-CPDI          IDGDYAANNGGWQWCASTGCDGQ-------PYFRIFNPVSQGEKFDPNGDFIRRWVPELR 425
Ec-CPDI          IDGDLAANNGGWQWAASTGTDAA-------PYFRIFNPTTQGEKFDHEGEFIRQWLPELR 424
Af-CPDIII        VDADPASNAANWQWVAGSGADAS-------PFFRIFNPILQGEKFDGDGDYVRRFVPELE 425
Ac-CPDIII        VDADLANNAASWQWVAGSGADAA-------PYFRIFNPVLQGEKFDPEGTYVRTYVPELK 427
Bd-CPDIII        VDADAGSNPANWQWVAGCGADAA-------PYFRVFNPQLQGEKFDPDGTYVRRWVPELQ 389
Ot-DASH          LDYDTASNWGNWCAAAGMTG--G--------RINRFNIAKQTKDYDPAGEYIKTWVKELA 465
Gv-DASH          IDYDVASNWGNWNYTAGVGNDAR--------GFRFFNILKQARDYDPDGAYVKHWLPELA 434
Ss-DASH          IDYDVCSNWGNWNYTAGIGNDAR--------DFRYFNIPKQSQQYDPQGTYLRHWLPELK 436
Dm-[6-4]Phr      LDQDWALNAGNWMWLSASAFFHQ--------YFRVYSPVAFGKKTDPQGHYIRKYVPELS 447
Os-[6-4]Phr      IDSDWAINNGNWMWLSCSSFFYQ--------YHRIYSPTSFGKKYDPNGNYIRHFIPVLK 457
Gv-[6-4]Phr      LDQDWSLNASNWMWLSASAFFNA--------YYRVYSPISFAKKYDPEGRYVRRYLPKLA 432
At-CPDII         IDGRDPSGYVGCMWSICGVHDQGWKERPVFGKIRYMNYAGCKRKFNVD-SYISYV-KSLV 471
Cf-CPDII         LDGRDPNGYAGVAWSIGGVHDRPWFERPVYGKIRYMNASGCARKFDVA-RYIARF-EEKK 448
Mm-CPDII         LDGRDPNGYAGIAWSIGGVHDRAWGEREVTGKIRYMSYEGCKRKFDVK-LYIEKY-SAL- 464
                 :*     .  .                      .  .     .. :    ::

Pc-Phr           SLNKKEVHNPALVGGL----FGV---ANYPSPIVDLSKSRERALAAFKAL-----PHRQP 475
Pk-CPDI          GLNKKEVHNPASAGGL----FGV---ADYPAPIVNLGASRERALAAFKNL-----PSRSS 475
Vc-CPDI          SVSSAYIHQPWTYPAV----N----SVLYPARLVDHKQEREVTLRLYKTA-----KG--- 469
Ec-CPDI          DVPGKVVHEPWKWAQK----AGV--TLDYPQPIVEHKEARVQTLAAYEAA-----RKGK- 472
Af-CPDIII        KLERKYIHKPFEAPKDALKKAGVELGKTYPLPIVDHGKARERALAAYAAV-----KKTT- 479
Ac-CPDIII        DVPARLIHQPWSA-PLELTRYGVRLGRDYPEPMVEHGAARDRALRAFEHT-----RKDLR 481
Bd-CPDIII        GLPAKLIHQPWQATPNELASAGVTLGKTYPQPIVDHARGRERALSAYAKI-----RKG-- 442
Ot-DASH          EVPAAYIADPNQAPRE----LRDRIGLNYPNKLALP------RRDFTEMGSPPGPRRGGG 515
Gv-DASH          GLPPARVHEPWKLLPVEQKRFGVRLGVDYPQPVVDLFQSAAANEAIYNAAWEHHHRK--- 491
Ss-DASH          NLPGDKIHQPWLLSATEQKQWGVQLGVDYPRPCVNFHQSVEARRKIEQMGVIA------- 489
Dm-[6-4]Phr      KYPATCIYEPWKASLVDQRAYGCVLGTDYPHRIVKHEVVHKENIKRMGAAYKVN-REVRT 506
Os-[6-4]Phr      DMPKEYIYEPWTAPLSIQKKANCIIGKDYPKPVVDHAIASKECKKMMGEAYASN-RLDDD 516
Gv-[6-4]Phr      RVPAEFIYEPWRAPLLVQKQAGCVVGRDYPDPIVDHEQAKACNLERMRLAYEQN-AKGG- 490
At-CPDII         SVTKKKRKA------------EEQLTRDSVDPKITIV----------------------- 496
Cf-CPDII         SM---------------------------------------------------------- 450
Mm-CPDII         ------------------------------------------------------------ 464

Pc-Phr           AADAL----------------HE------------------------        482
Pk-CPDI          MGEVH----------------E-------------------------        481
Vc-CPDI          -----------------------------------------------        469
Ec-CPDI          -----------------------------------------------        472
Af-CPDIII        -----------------------------------------------        479
Ac-CPDIII        SAG--------------------------------------------        484
Bd-CPDIII        -----------------------------------------------        442
Ot-DASH          GGGRG----------------RGRPGGSTPNRGTKARVASVYDTVYG        546
Gv-DASH          ----R----------------RGQPRSRT------------------        500
Ss-DASH          -----------------------------------------------        489
Dm-[6-4]Phr      GKEEESSFEEKSETSTSGKRKVRRATGSAPKRKR-------------        540
Os-[6-4]Phr      KPDKGK-------SSNSSRRKLSAGSQVTPNSSKTKQLKRSS-----        551
Gv-[6-4]Phr      -----------------------------------------------        490
At-CPDII         -----------------------------------------------        496
Cf-CPDII         -----------------------------------------------        450
Mm-CPDII         -----------------------------------------------        464
```

Fig. S5. Multiple sequence alignment of Pc-Phr with representative photolyase proteins. The deduced amino acid sequences of Pc-Phr from *Pseudomonas cichorii* JBC1 (NCBI ID: PCH70_09020) are aligned with representative photolyases in multiple classes using Clustal Omega. The aligned sequences include class I CPD photolyases: *P. koreensis* (Pk-CPDI, RVD74662), *Vibrio cholerae* O1 (Vc-CPDI, Q9KNA8), and *Escherichia coli* K-12 (Ec-CPDI, P00914); class II CPD photolyases: *Arabidopsis thaliana* (At-CPDII, Q9SB00), *Chlorobium ferrooxidans* DSM13031 (Cf-CPDII, Q0YV00), and *Methanosarcina mazei* (Mm-CPDII, Q8PYK9); class III CPD photolyases: *Agrobacterium fabrum* C58 (Af-CPDIII, AAK87020), *Azorhizobium caulinodans* ORS 571 (Ac-CPDIII; BAF88822), and *Bradyrhizobium diazoefficiens* USDA 110 (Bd-CPDIII, BAC50575); (6–4) Photolyases: *Drosophila*

melanogaster (Dm-[6–4]Phr, Q24281), *Oryza sativa* (Os-[6–4]Phr, Q0E2Y1), and *Gloeobacter violaceus* (Gv-[6-4]Phr, Q7NJT3); and Cry-DASHs: *Gloeobacter violaceus* PCC7421 (Gv-DASH; Q7NMD1), *Synechocystis* sp. PCC6803 (Ss-DASH; P77967), and *Ostreococcus tauri* (Ot-DASH, Q5IFN2). The DNA photolyase and FAD-binding domains are highlighted in yellow and green, respectively. Conservation of amino acid residues is indicated as follows: "*" (fully conserved), ":" (strongly conserved), "." (weakly conserved), and space (non-conserved). The conserved tryptophan residues involved in the electron transfer chain (W382–W359–W306 in Ec-CPDI) are marked with red triangles and framed in red.

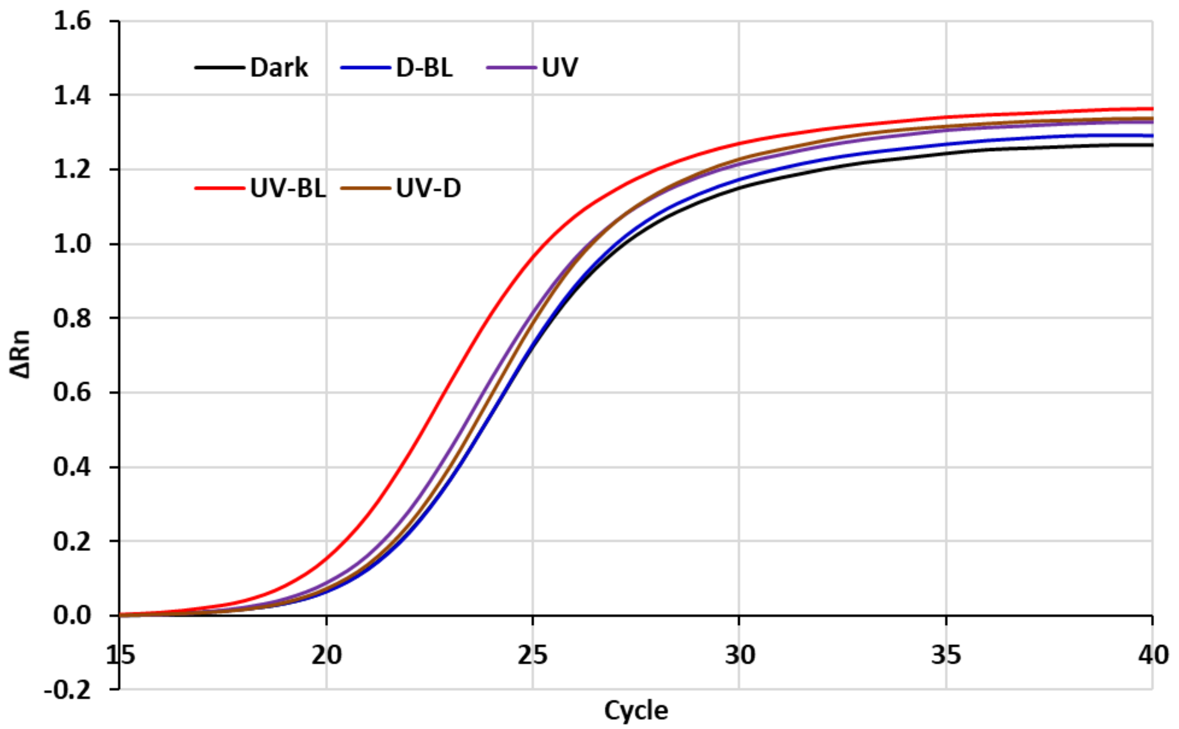

Fig. S6. Effects of blue light and UV-C on the amplification curves of *phr* gene. PcJBC1 was cultured in LB agar under dark condition until the exponential growth phase is reached. Cells were then exposed to UV-C (254 nm, UV) for 45 s or maintained in darkness (Dark). Following treatment, the cultures were incubated for 15 min either under BL (458 nm at 2.5 μmol/m^{2}s; UV-BL and D-BL, respectively) or in the dark condition (UV-D). The total RNA was extracted, and the *phr* expression was analyzed using RT-qPCR.

Tables S1. Primers utilized in this study

Name	Primer Sequence (5'→3')[a]	Use
HindIII-PHR-F	ATGAAGCTTTCACTCATGGAGCG	Cloning *phr* into pET28a vector
BamHI -PHR-R	ATTGGATCCATGCAACTGTTTTGGC	
Sg_PHR_F	GTGGAATGACGGAGAATTCGAGAC	Generation of target sequence for *phr*
Sg_PHR_R	AAACGTCTCGAATTCTCCGTCATT	
R-Amp	CAAGGCGAGTTACATGATCCCCCA	Checking presence of pACRISPR-sgRNA-lov1
PHR_Us_F	TTTTGAGATCTGTCCATACCCATGGTCTAGACC GAGCTGGAAAAGCGCTGG	Amplification of upstream region of *phr* gene
PHR_Us_R	CGTTGCAGGAATTCACTCATGTGAAATCCTTAA ATGAGTCCGAGCCTG	
PHR_Ds_F	GACTCATTTAAGGATTTCACATGAGTGAATTCC TGCAACGCTTCGCCCATG	Amplification of downstream region of *phr* gene
PHR_Ds_R	TCTGAATGGCGGGAGTATGAAAAGTCTCGAGG AGCAACTCTTTTGCCAGG	
PHR_HindIII_500Us_F	CATGAAGCTTCCGAGCTGGAAAAGCGCTGG	Cloning *phr* and its promoter regions into pUCP18
PHR_BamHI_R	GTATAGGATCCTCACTCATGGAGCGCATCCG	
pPHR_BamHI_R	GTATAGGATCCGTGAAATCCTTAAATGAGTC	
M13F(-40)	GTTTTCCCAGTCACGAC	Verification of the *phr*-complemented strain (JBC1$^{\Delta phr}$+pphr)
M13pUC-R	GCGGATAACAATTTCACACAG	
PHR_F	TTCCGCATCTTCAATCCGCT	RT-PCR for *phr*
PHR_R	CAGGTCTACAATCGGGGACG	
RecA_F	GCCGTGAAAGAAGGCGATGA	RT-PCR for *recA*
RecA_R	CAAAGCCATGCAACACAGCC	
RpoD-F	CGGCCTGAAAATCGCCGAGATC	RT-PCR for *ropD*
RpoD-R	CAGACGCAAGTTGGCTTCAACC	
UvrA_F	CGATGAAGACGCTATCCGCC	RT-PCR for *uvrA*
UvrA_R	CGCTTGGCCGGTACTTCAAT	

[a]Restriction enzyme sites are underlined

Table S2. Characteristics of the photolyases encoding genes of *Pseudomonas cichorii* JBC1 and other organisms

Strains	NIBI ID number	Length (bp)	Identities (aa)[a]	MW (kDa)[b]	pI[b]	GRAVY[b]
Pc-Phr	PCH70_09020	1449	100.0%	54.49	6.85	-0.364
Pk-CPDI	RVD74662	1449	79.25%	54.08	6.75	-0.286
Ec-CPDI	b0708	1419	44.59%	53.67	6.74	-0.395
Vc-CPDI	VC_A0057	1410	41.47%	54.36	6.29	-0.390
At-CPDII	Q9SB00	1491	20.45%	57.05	9.03	-0.436
Cf-CPDII	Q0YV00	1353	23.06%	52.00	8.56	-0.477
Mm-CPDII	Q8PYK9	1395	24.04%	53.16	5.74	-0.409
Ac-CPDIII	BAF88822	1455	37.26%	53.16	8.38	-0.234
Af-CPDIII	AAK87020	1440	36.18%	53.89	9.01	-0.462
Bd-CPDIII	BAC50575	1329	37.39%	48.92	9.73	-0.429
Dm-(6-4)Phr	Q52Z99	1803	28.94%	67.08	9.51	-0.422
Os-(6-4)Phr	Q0E2Y1	1656	29.58%	62.26	8.87	-0.453
Gv-(6-4)Phr	Q7NJT3	1473	28.99%	55.77	6.82	-0.391
Gv-DASH	Q7NMD1	1503	30.19%	57.26	7.82	-0.391
Ot-DASH	Q5IFN2	1641	25.66%	60.98	8.72	-0.461
Ss-DASH	P77967	1470	28.18%	57.04	8.82	-0.531
Vc-FeS-BC	Q9KLD7	1551	NSS	59.43	7.06	-0.316
Cs-FeS-BCP	Q3IXP1	1527	NSS	58.04	6.75	-0.368
Af-FeS-BCP	A9CH39	1524	NSS	57.86	6.05	-0.387
Ds-NewPHL	A8LJA9	1218	29.81%	44.79	6.68	-0.303
Mm-NewPHL	M7YZC8	1131	29.78%	40.86	6.43	-0.131
El-NewPHL	Q2N8F4	1224	30.60%	45.04	6.07	-0.413
Rc-NewPHL	B6IPR5	1455	33.56%	53.03	7.26	-0.256

[a]The deduced amino acid sequence of Pc-Phr proteins from *P. cichorii* JBC1 was compared with Class I photolyases: *Pseudomonas koreensis* (Pk-CPDI), *Escherichia coli* K-12 (Ec-CPDI), *Vibrio cholerae* O1 (Vc-CPDI); Class II photolyases: *Arabidopsis thaliana* (At-CPDII), *Chlorobium ferrooxidans* DSM13031 (Cf-CPDII); *Methanosarcina mazei* (Mm-CPDII); Class III photolyases: *Agrobacterium*

fabrum C58 (Af-CPDIII), *Azorhizobium caulinodans* ORS 571 (Ac-CPDIII), *Bradyrhizobium diazoefficiens* USDA 110 (Bd-CPDIII); (6-4)Photolyases: *Drosophila melanogaster* (Dm-(6-4)Phr), *Oryza sativa* (Os-(6-4)Phr), *Gloeobacter violaceus* (Gv-(6-4)Phr); Cry-DASHs: *Gloeobacter violaceus* PCC7421 (Gv-DASH), *Synechocystis* sp. PCC6803 (Ss-DASH), *Ostreococcus tauri* (Ot-DASH); FeS-BCPs: *Vibrio cholerae* (Vc-FeS-BCP), *Cereibacter sphaeroides* (Cs-FeS-BCP), *Agrobacterium fabrum* (Af-FeS-BCP); and NewPHLs: *Dinoroseobacter shibae* (Ds-NewPHL), *Methylobacterium mesophilicum* (Mm-NewPHL), *Erythrobacter litoralis* (El-NewPHL), and *Rhodospirillum centenum* (Rc-NewPHL) using the NCBI BLAST. NSS, No significant similarity.

[b]The moleculer weight (MW, kDa), isoelectric point (pI), and the grand average of hydropathy value (GRAVY) were predicted using ExPaSy-Protparam.

Table S3. Original survival percentage data (%) from the experiment evaluating the effects of Pc-Phr and blue light on the survival of *Pseudomonas cichorii* JBC1

Bacterial strains	Conditions*			
	Dark	BL2.5	BL5.0	BL7.5
PcJBC1	100	100	100	74.3
PcJBC1	100	99.8	98.8	70.9
PcJBC1	100	99.6	99.5	61.4
JBC1$^{\Delta phr}$	100	100	55.6	33.3
JBC1$^{\Delta phr}$	100	99.6	58.3	34.0
JBC1$^{\Delta phr}$	100	99.3	64.1	39.4
JBC1$^{\Delta phr}$+p18	100	100	50.5	33.7
JBC1$^{\Delta phr}$+p18	100	99.3	48.5	32.0
JBC1$^{\Delta phr}$+p18	100	99.3	57.6	25.5
JBC1$^{\Delta phr}$+pphr	100	100	80.0	45.5
JBC1$^{\Delta phr}$+pphr	100	99.8	85.0	41.0
JBC1$^{\Delta phr}$+pphr	100	99.5	85.1	43.7

*Bacterial cells were spread on LB plates and incubated at 28 °C under BL at intensities of 2.5, 5, and 7.5 µmol/m^2·s and dark conditions for 48 h.

Table S4. Effects of blue light and UV-C on Ct values in RT-PCR analysis of *phr* and *rpoD* genes in *Pseudomonas cichorii* JBC1.

Target gene	Conditions*				
	Dark	D-BL	UV	UV-BL	UV-D
rpoD	22.97 ± 1.12	22.98 ± 1.18	22.95 ± 1.15	22.93 ± 1.13	22.96 ± 1.12
phr	20.04 ± 1.02	20.00 ± 1.25	19.60 ± 1.09	18.73 ± 0.91	19.95 ± 1.09

* The bacterium was cultured in LB agar under dark condition until the exponential growth phase is reached. Cells were then exposed to UV-C (254 nm) for 45 s or maintained in darkness (Dark). Following treatment, the cultures were incubated for 15 min either under BL (458 nm at 2.5 μmol/m^2s; UV-BL and D-BL, respectively) or in the dark condition (UV-D). The total RNA was extracted, and the *phr, rpoD* expression was analyzed using RT-qPCR. The data represent the mean ± SD of five independent biological replicates.
